# Wild again: recovery of a beneficial *Cannabis* seed endophyte from low domestication genotypes

**DOI:** 10.1186/s40168-024-01951-5

**Published:** 2024-11-15

**Authors:** Carolina Lobato, João Machado de Freitas, Daniel Habich, Isabella Kögl, Gabriele Berg, Tomislav Cernava

**Affiliations:** 1 Institute of Environmental Biotechnology, Graz University of Technology, Petersgasse 12, 8010 Graz, Austria; 2 Institute for Signal Processing and Speech Communication, Graz University of Technology, Inffeldgasse 16C/EG, Graz, 8010 Austria; 3 Leibniz Institute for Agricultural Engineering and Bioeconomy, Max-Eyth-Allee 100, 1446 Potsdam, Germany; 4 Institute for Biochemistry and Biology, University of Potsdam, Karl-Liebknecht-Str. 24-25, Potsdam-Golm, 14476 Germany; 5 School of Biological Sciences, Faculty of Environmental and Life Sciences, Highfield Campus, Southampton, SO17 1BJ UK

**Keywords:** Bacterial communities, Plant breeding, Plant fitness, Plant–microbe interactions, Plant microbiome, Seed endophytes

## Abstract

**Background:**

Beyond carrying the plant embryo, seeds harbour intricate microbial communities whose transmission across successive plant generations can significantly influence the ecological and evolutionary dynamics of plant–microbe symbioses. The process of plant domestication has potential repercussions in genes involved in plant-microbiome interactions. However, the extent to which breeding can impact the seed microbiome is sparsely explored. *Cannabis* is a high-value crop but sparsely subjected to agricultural innovations established in other crop species during the last century. Here, we conduct a large-scale analysis of the bacterial seed microbiome of *Cannabis* across different domestication grades and investigate the potential of seed-associated endophytes as plant growth-promoting agents under both controlled and field conditions.

**Results:**

Analysis of *Cannabis* seed endophyte composition and diversity across 46 plant genotypes revealed 813 different bacterial genera with a predominance of Gammaproteobacteria, Bacilli, Actinobacteria and Alphaproteobacteria but a genotype-specific microbiome. The assessment of domestication and breeding on microbial assembly revealed a higher bacterial diversity in low domestication genotypes (Shannon index, H′: 1.21 vs. 1.05) and a higher homogeneity in bacterial composition caused by line development. Further, a seed bacterial isolate (*Bacillus frigoritolerans* C1141) associated with low domestication genotypes, and with genes associated with bio-fertilization, bioremediation and phytohormone production, increased plant growth by 42.3% at the time of harvest, under field conditions.

**Conclusion:**

This study addresses critical knowledge gaps related to the assembly of the *Cannabis* seed-endophytic microbiome. It reveals that *Cannabis* breeding is linked to alterations of seed microbial communities, which potentially led to the loss of bacteria with functional significance. These results highlight the importance of preserving seed microbiomes in plant breeding to support sustainable plant health and growth enhancement in *Cannabis*.

Video Abstract

**Supplementary Information:**

The online version contains supplementary material available at 10.1186/s40168-024-01951-5.

## Background

Plants host microbial communities that are crucial for their growth and vitality [1]. These microbes play multifaceted roles from nutrient mobilization to stress mitigation and enhanced defence against pathogens [2]. Seeds, pivotal in the continuum of plant existence, serve not merely as vessels of plant propagation but as reservoirs of microbial diversity, integral to plant success. Seed-associated microorganisms wield their influence on plants by steering the course of seed germination, preservation, and early development [3]. Furthermore, seed endophytes can be transferred across generations, fostering symbiotic and mutualistic relationships with plants that lay the foundation for robust plant establishment [4–6]. Consequently, seeds emerged as promising sources for the isolation of beneficial microorganisms, as well as targets for microbiome-based breeding strategies [7, 8]. However, a comprehensive understanding of seed microbiome structures and functions remains limited, particularly within the *Cannabis* genus.

*Cannabis*, a herbaceous annual plant belonging to the *Cannabaceae* family, boasts a rich history of domestication alongside significant potential for future utilization [9]. Originating in eastern Asia, *Cannabis* has transcended borders to become a cosmopolitan crop valued for its versatility in providing fibre, oil, food, and bioactive compounds for medicinal as well as recreational purposes. The human-driven selection across diverse regions has contributed to its extensive diversification [10], resulting in predominantly hybrid genotypes today [11]. Notably, the accumulation of tetrahydrocannabinol (THC), which has psychoactive properties, in the trichomes of female flowers, has posed challenges in resource management and genetic enhancement of *Cannabis* [12]. Recent trends favour cannabidiol (CBD)-rich genotypes for therapeutic purposes, reflecting a projected global market value of US $75.09 billion by 2029 [13]. However, scaling up *Cannabis* production brings forth challenges, including heightened susceptibility to fungal pathogens and bacterial infections in greenhouse settings and under field conditions [14]. Additionally, mitigating the environmental impact of cultivation practices is essential to align with climate objectives while meeting production demands [15].

Through the processes of plant domestication and breeding, humans have tailored crops to specific quality traits, reshaping the very essence of plant physiology, morphology, and genetic makeup, which has resulted in a discernible reduction in allelic diversity [16]. These processes might have potential repercussions for genes involved in plant-microbiome interactions and thus affect key traits in plant anatomy, immunity or production of secondary metabolites [17]. For instance, alterations in root morphology and exudate profiles of different plant genotypes can alter the selective recruitment of microorganisms from soil [18–22]. Under domestication, seeds often exhibit the most striking phenotypic alterations, emerging as an optimal domain for exploring those effects on microbial communities [23]. Still, little is known about the intricate nuances of these processes on seed microbial assembly.

Previous studies have outlined the differences in microbial communities of different *Cannabis* genotypes across plant compartments [24–27]. From those, only a recent study described the variations in bacterial diversity in the *Cannabis* seed microbiome, however, limited to genotypes with low THC content [28]. Furthermore, the question of whether *Cannabis* breeding influenced microbial assembly in seeds is left unanswered. Here, we conducted an extensive characterization of the *Cannabis* bacterial seed microbiome across 46 genotypes with different domestication grades and chemotypes, to cover the high variability of the genus. Seed accessions from early domestication genotypes obtained through natural and artificial selection were included; they are represented by landraces and selected lines. We also included modern genotypes that were obtained through artificial selection; they are represented by accessions classified as cross hybrids and inbred lines. We hypothesized that the heterogeneous genetic background of *Cannabis* reflects on its seed microbiome. The potential of *Cannabis* seed endophytes as plant growth-promoting (PGP) and biocontrol agents against different pathogenic fungi has been previously shown [29]. In this work, we tested the potential of *Cannabis* bacterial endophytes, associated with low domestication genotypes, in promoting growth of *Cannabis* under controlled and field conditions. We further searched for potential PGP functions in the best-performing bacterium, using whole genome sequencing (WGS). The overall goal of this work is twofold: to comprehensively explore the *Cannabis* seed microbiome and assess whether this was potentially influenced by domestication, as a strategy for harnessing beneficial seed-endophytic microorganisms that can be used as a sustainable alternative for *Cannabis* cultivation.

## Materials and methods

### Metabarcoding analysis

#### Sample collection

In this study, we comprehensively analysed the bacterial seed microbiome of 46 distinct *Cannabis* genotypes, sourced from multiple institutions and seed companies across Europe. Seeds were obtained from the Crop Research Institute (CRI) in Prague, Czech Republic (*n* = 2); the Green House Seed Co. in Amsterdam, Netherlands (*n* = 2); the Leibniz Institute for Plant Genetics and Crop Plant Research (IPK) in Gatersleben, Germany (*n* = 27); Hanfama GmbH in Graz, Austria (*n* = 6); Hanfland GmbH in Hanfthal, Austria (*n* = 1), Botanical Garden of the University of Graz, Austria (*n* = 1); the Latvian State Forest Research Institute (LVMI) Silava in Salaspils, Latvia (*n* = 1); and Sensi Seeds in Amsterdam, Netherlands (*n* = 6). The sampled genotypes encompassed a spectrum of reproductive types, including dioecious (*n* = 27), monoecious (*n* = 9), and sub-dioecious populations (*n* = 2), as well as varying chemotypes, ranging from low cannabinoid (*n* = 33) to THC- or CBD-enriched genotypes (*n* = 9). Furthermore, the samples reflected various grades of domestication, classified as landraces (*n* = 13), defined for the purpose of this paper as genetically heterogeneous genotypes with minimal domestication [30], selected lines derived from landrace populations (*n* = 4), a variety of cross hybrids bred from diverse *Cannabis* genotypes (*n* = 11), and inbred lines obtained from cross hybrids (*n* = 5), along with one segregating hybrid and two feminized hybrids obtained by selfing (hybrid S1). Here, we classify seed accessions from early domestication genotypes, i.e. landraces and selected lines, as low domestication genotypes, and modern genotypes, i.e. cross hybrids and inbred lines, as high domestication genotypes. We have included several key *Cannabis* accessions that have significantly contributed to the development of modern genotypes used for industrial purposes in Europe, the USA and Canada, such as Schurig, Havelländer, Carmagnola and Fibrimon. Comprehensive details regarding the *Cannabis* genotypes utilized in this study are provided in Table S1.

#### Amplicon library preparation and sequencing

Seeds underwent a pretreatment process to eliminate non-endophytic microorganisms. Initially, seeds were soaked in sterile deionized water for 4 h on a shaker at 125 rpm. Subsequently, surface sterilization was conducted using a 4% solution of sodium hypochlorite (NaClO) for 5 min with agitation, followed by three 5-min rinses in sterile water. The efficacy of sterilization was confirmed by inoculating 100 μL of the final rinse water onto nutrient agar II (NA II) plates. Seeds were then germinated under sterile conditions until the emergence of radicle and cotyledons. Soaking and germinating seeds can provide a better picture of the endophytic communities by allowing possible rare microorganisms to be activated and multiply to detectable levels [31]. Ten replicate samples, comprising two seedlings each, were obtained for each genotype, except for *ID* = C86 (8 replicates) and *ID* = C49 (9 replicates), resulting in a total of 457 samples. This number of replicates allows to sufficiently address the intra-genetic variety of seed-propagated *Cannabis* genotypes [32]. Subsequently, samples were ground with a pestle in 4 mL of 0.85% NaCl under sterile conditions, and the resulting homogenate was pelleted for further processing. Total DNA extraction was performed using the FastDNA™ SPIN Kit for Soil and the FastPrep Instrument (MP Biomedicals, Santa Ana, CA, USA), following the manufacturer’s instructions. DNA quality was assessed using a NanoDrop 2000 (Thermo Scientific, Wilmington, DE, USA), and samples were stored at − 20 °C for subsequent PCR reactions. The 16S rRNA gene V4 hypervariable region was amplified using the 515f/806r primer pair (515f: 5′-GTGYCAGCMGCCGCGGTAA-3′; 806r: 5′-GGACTACNVGGGTWTCTAAT-3′) [33], with peptide nucleic acid (PNA) clamps employed to block amplification of plastid and mitochondrial 16S rRNA genes during PCR [34]. PCR reactions were performed in a total volume of 30 µL and in three technical replicates using 5 × Taq-&GO Ready Mix (MP Biomedicals, Illkirch, France), 0.2 µM of each primer, 1.5 µM PNA mix (1:1), PCR-grade water, and 3 µL of DNA template. PCR amplification was conducted for 35 cycles after initial denaturation at 96 °C for 5 min, with denaturation at 96 °C for 30 s, PNA annealing at 78 °C for 5 s, primer annealing at 54 °C for 30 s, and elongation at 74 °C for 30 s, followed by a final elongation at 72 °C for 10 min. Amplification products were purified using the Wizard SV Gel and PCR Clean-Up System (Promega, Madison, WI, USA) and pooled at equimolar concentrations in three different pools, each including negative controls for DNA extraction and PCR, and PCR products amplified from the ZymoBIOMICS Microbial Community DNA Standard (Zymo Research, Orange, CA, USA) to infer on the bias and errors introduced by sequencing library preparation. Barcoded Illumina libraries were generated and subjected to paired-end sequencing on an Illumina NovaSeq Instrument (Novogene Co., Ltd., UK). The resulting 16S rRNA gene amplicon dataset was deposited in the European Nucleotide Archive (ENA; https://www.ebi.ac.uk/ena) under the accession number PRJEB64469.

#### Bioinformatic processing

Initial processing involved quality checking and demultiplexing of pair-end reads using Cutadapt v4.2 [35]. Subsequently, the DADA2 algorithm within QIIME2 v2023.5 was employed for read quality filtering, denoising, read merging, and generation of representative sequences as amplicon sequence variants (ASVs) and the feature table [36, 37]. Taxonomic classification was performed utilizing the VSEARCH algorithm implemented in QIIME2, with the Silva v138 database utilized as the reference for bacterial 16S rRNA gene sequences [38, 39]. Following classification, unassigned and nontarget sequences, including chloroplasts, mitochondria, and archaea, were removed from further analysis. Additionally, samples with fewer than 1000 reads (*n* = 6) were excluded to ensure a quality-filtered dataset. Contaminant ASVs were identified and eliminated from the dataset using decontam [40] in R v4.2.3 [41] based on prevalence utilizing the fisher method. Ultimately, a total of 36,996,902 high-quality reads, with a mean of 82,033 reads per sample, were retained, resulting in 5297 bacterial ASVs across 451 samples derived from 46 distinct *Cannabis* genotypes.

#### Amplicon data analysis

Data manipulation, statistical analysis, and representation were conducted using R v4.2.3 [41] with tidyverse unless otherwise specified. The feature table and taxonomic information were analysed using phyloseq [42]. To ensure comparability, a dataset rarefied to 5607 reads per sample was obtained using rarefy_even_depth from phyloseq on the quality-filtered dataset (Fig. S1). The rarefied dataset was utilized to calculate observed ASV richness and Shannon *H′* index. Normality of alpha-diversity measures was assessed using shapiro.test from stats, followed by analysis of variance using kruskal.test, modelling these variables as a function of *Cannabis* genotype and domestication grade. Pairwise comparisons were calculated using pairwise.wilcox.test with the Bonferroni adjustment method. A heat tree was generated using metacoder [43] down to the genus level with a minimum relative abundance cut-off of 1e-5 for visualization purposes. For beta-diversity calculations, phyloseq_transform_css from metagMisc [44] was employed to normalize counts using cumulative sum scaling (CSS) transform on the quality-filtered dataset. The Bray–Curtis dissimilarity matrix was then calculated using vegdist and subjected to permutational multivariate analysis (PERMANOVA, 999 permutations) with adonis2 in vegan [45] to test differences in microbial communities based on *Cannabis* genotype, chemotype and domestication grades. Pairwise comparisons were performed using pairwise.adonis in pairwiseAdonis, with false discovery rate correction [46]. Results were projected with UMAP based on Bray–Curtis dissimilarity [47], and silhouette information was extracted from clustering using cluster [48]. The most abundant bacteria (detection > 0.05) were plotted within each genotype with plot_composition from microbiome [49] and the core microbiome across different domestication grades determined with core_members. To further explore the intricate connection between the seed microbiome and *Cannabis* domestication, we modelled our data with machine learning (ML) using multi-class classification with Gradient Boosted Trees (XGBoost) [50] via tidymodels [51]. Using the trained model, we explained the relative contribution of each ASV to the ML model’s predictions using a feature importance score based on SHAP (SHapley Additive exPlanations) values estimated with shapviz [52]. Features were preprocessed based on a Kruskal–Wallis test (*α* = 0.05) relative to the domestication grade and abundance data, normalized for training using the CSS transform. To evaluate the predictive performance of the models, we employed five repetitions of tenfold cross-validation and assessed the area under the receiver operating curve (AUC). Finally, we tailored our feature importance score to prioritize abundant taxa. First, we kept only the SHAP values of the overly abundant taxa associated with the domestication level; then, we estimated the average SHAP values of each of the taxa, weighted by the min–max normalized abundance values. Detailed model hyperparameters are provided in Table S2. 

### Preselection of bacterial strains

#### Bacterial isolation and identification

Bacterial isolates were obtained from surface-sterilized, germinated seeds of *Cannabis sativa* L. as detailed above and subsequently cultured on nutrient agar (NA II, SIFIN, Berlin, Germany) amended with nystatin (25 μg/mL). For DNA extraction, bacterial cells underwent thermal lysis at 100 °C for 10 min in a buffer (pH 8) containing 10-mM Tris–Cl, 1 mM EDTA, and 1% Triton X-100, followed by rapid cooling to − 20 °C. DNA integrity was verified using a NanoDrop 2000 spectrophotometer (Thermo Scientific, Wilmington, DE, USA). The full-length 16S rRNA gene was amplified using primers 27f (5′-AGAGTTTGATCMTGGCTCAG-3′) and 1492r (5′-TACGGYTACCTTGTTACGACTT-3′). PCR conditions included an initial denaturation at 95 °C for 5 min, followed by 35 cycles of denaturation at 95 °C for 30 s, annealing at 61 °C for 30 s, and elongation at 72 °C for 90 s, concluding with a final elongation at 72 °C for 10 min. PCR products were purified with the Wizard SV Gel and PCR Clean-Up System (Promega, Madison, WI, USA) and sequenced via Sanger sequencing (LGC Genomics, Berlin, Germany). Sequences were submitted to manual quality filtering with BioEdit 7.7 [53] and analysed using the NCBI BLAST + blastn megablast tool hosted on Galaxy (https://usegalaxy.org/). Only sequences with 100% match to the V4 region of the reference metabarcoding sequences were considered for the identification of the selected bacterial markers.

#### Bacterial treatments and plant-growing conditions

Selected bacterial strains were cultured individually in Nutrient Broth II (NB II, Difco) at 30 °C overnight until reaching the exponential phase of growth. Cultures were purified through two rounds of centrifugation at 5000 rpm for 10 min, and the resulting bacterial pellets were resuspended in a 0.85% NaCl solution, whereas bacterial concentrations were adjusted to 1 × 10^8^ CFU/mL. Seeds of the *Cannabis* genotype Fedora 17, an inbred line, were exposed to the different bacterial suspensions for 4 h under agitated conditions at room temperature, followed by germination in sterile CYG germination pouches (MEGA International), each soaked with 15 mL of sterile dH_2_O. Germinating seeds were kept in dark conditions for 5 days and subsequent 2 days in a greenhouse under a 16-h light cycle at 25 °C. Control seeds were incubated in sterile 0.85% NaCl. The experimental design included three technical replicates per treatment, each comprising 40 seeds. To evaluate the plant growth-promoting effects of the different treatments, data analysis was performed in R v4.2.3 using compare_means from ggpubr [54], applying a nonparametric Wilcoxon rank-sum test with Bonferroni correction for multiple comparisons. The distribution of the variables was assessed using the shapiro.test from stats [41]. For categorical outcomes, the chisq.test with Bonferroni correction for multiple comparisons was employed to analyse the differences between treatments and control.

### Field trials

#### Bacterial treatments and field design

The field trial was conducted at the Experimental Station for Special Cultures (Versuchsstation für Spezialkulturen) in Wies, Austria, within a 187.5 m^2^ plot divided into three blocks with 1.5 m distance between them. The experimental design included treatment with *Bacillus frigoritolerans* C1141 (ASV 3055), isolated from the endosphere of *Cannabis* seeds, and *Serratia plymuthica* RR2-5–10, isolated from the rhizosphere of oilseed rape cultivated in weakly loamy sand near Rostock, Germany [55]. The latter has been documented for its beneficial effects in various plant systems [56]. Each block consisted of 30 *Cannabis* plants originated from cuttings of Eletta Campana, a cross hybrid, totalling 90 plants for the experiment. Bacterial stock solutions were prepared from overnight cultures; on the planting day, these cultures were centrifuged, and the cell pellets were resuspended in 0.85% NaCl. These preparations were then diluted to a final concentration of 3 × 10^7^ CFU/mL in the field. The treatment protocol involved immersing the roots of the *Cannabis* plants in the bacterial suspensions for 5 min before planting, followed by the addition of 50 mL of the suspension to the rhizosphere of each plant. Control plants were treated with the same volume of sterile water. The soil at the Experimental Station for Special Cultures was loamy with a pH of 6.6, 4.4% of organic matter, 18 cmol + /kg calcium, a water retention capacity of 23.7% of its weight and with the following nutrient content: > 200 mg/1000 g of plant-available phosphorus, 526 mg/1000 g of plant-available potassium and 183 mg/100 g of plant-available magnesium. The plot was tilled using Geohobel (Rath) and irrigated before planting. No irrigation, fertilizers, or pesticides were applied during the trial. The concentrations of NO_3_ and NH_4_ in the soil were 7 kg/ha and 24 kg/ha, respectively. Recorded climatic conditions from June to September showed average temperatures ranging from 13.7 to 25.9 °C, with total rainfall measuring 155.7 mm. August was the wettest month with 258.4 mm of rain, while September recorded the least precipitation at 49.4 mm. Growth metrics, including plant height and stalk diameter, were collected monthly and final biomass assessments at the study’s conclusion.

#### Statistical data analysis for plant growth parameters

To evaluate the plant growth-promoting effects, data analysis was performed in R. Differences in growth outcomes between treatments were determined using the compare_means from ggpubr [54], applying a nonparametric Wilcoxon rank-sum test with Bonferroni correction for multiple comparisons. The distribution of the variables was assessed using the shapiro.test from stats [41].

### Whole genome sequencing

#### DNA extraction and PacBio sequencing

DNA from *B. frigoritolerans* C1141 was extracted from overnight cultures in NB II (*OD*_600_ = 2.46) for WGS. The extraction was performed using the MasterPure Complete DNA and RNA Purification Kit (Epicentre, Madison, WI, USA), following the manufacturer’s instructions. Additional steps were incorporated for enhanced lysis efficiency, including supplementation of the lysis buffer with lysozyme and transferring of the samples into a Lysing Matrix E tube containing 1.4-mm ceramic spheres, 0.1-mm silica spheres, and one 4-mm glass sphere for mechanical lysis using a FastPrep-24 instrument (MP Biomedicals) at 6 m/s for 25 s, twice, keeping samples 1 min on ice between repetitions. The concentration of extracted DNA was measured using the Qubit dsDNA HS Assay Kit on a Qubit 4 Fluorometer (Thermo Fisher Scientific, Waltham, MA, USA). Subsequently, the DNA was subjected to PacBio sequencing on a PacBio Sequel II/IIe platform after SMRTbell library construction (Novogene Co., Ltd., UK).

#### Genome processing and mining

The continuous long-read sequencing (CLR) single reads obtained (*n* = 370,273) were employed for de novo assembly using Flye v2.9.1 [57], resulting in a closed circular chromosome spanning 5,620,668 bp. To assess genome completeness and contamination levels, the assembled genome was evaluated using CheckM v1.2.2 [58], estimating values of 98.81% and 1.42%, respectively. Taxonomic classification was verified using GTDB-Tk v1.4.1 [59], and gene annotation was performed using DRAM v1.2.2 [60] resulting in the identification of 5518 predicted protein coding sequences. Subsequently, amino acid sequences generated by DRAM were utilized to annotate bacterial PGP functions on PlaBAse v.1.02 employing strict mode blast + hmmer. The assembled genome, along with the associated annotations, was deposited in the National Center for Biotechnology Information (NCBI; https://www.ncbi.nlm.nih.gov/) under accession number PRJNA1113337.

## Results

### Diversity of *Cannabis* seed endophytes changes with domestication grade

**Fig. 1:**
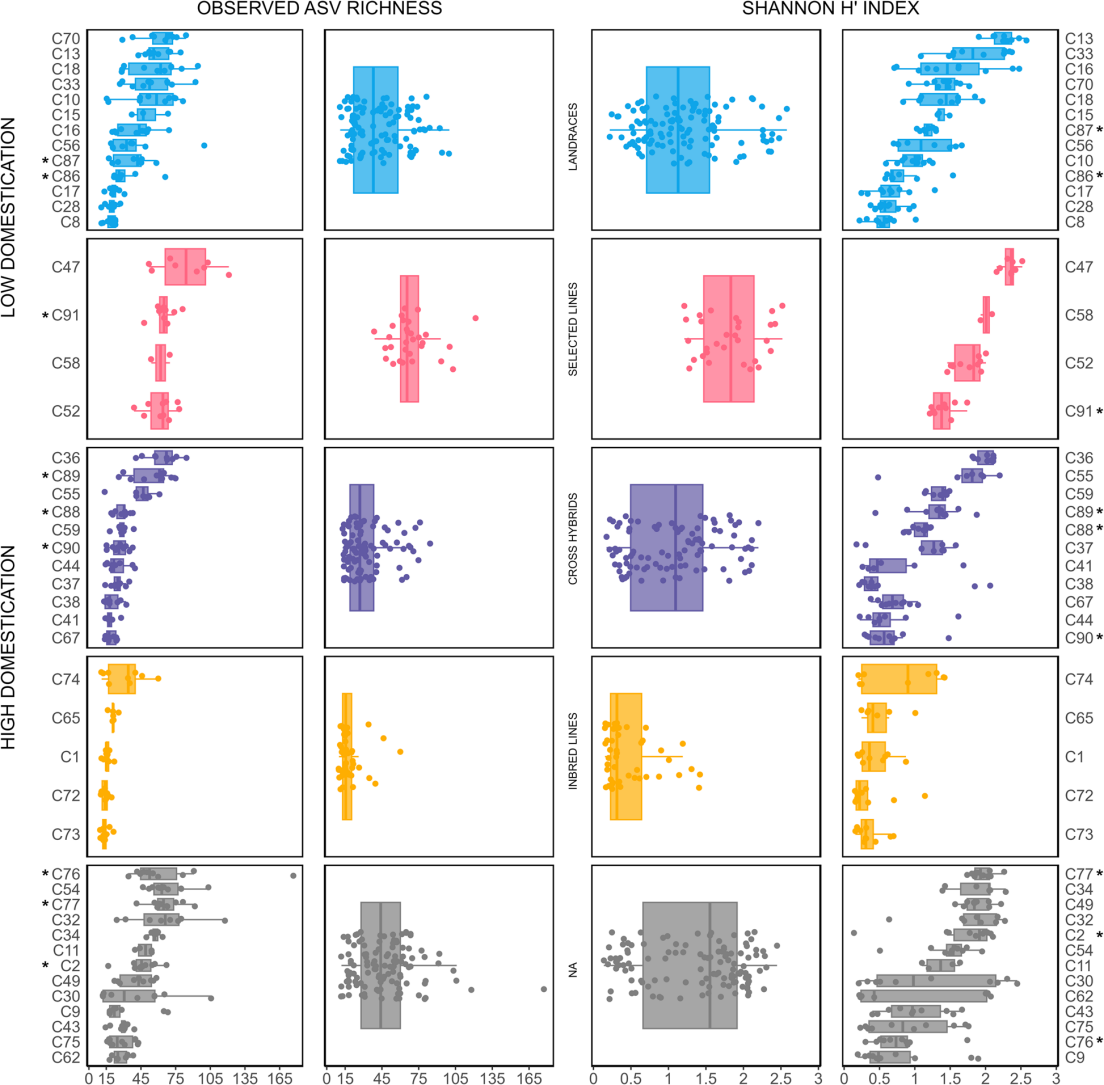
Observed amplicon sequence variants (ASVs) richness and Shannon *H′* index were used to assess seed microbiome diversity within and across *Cannabis* genotypes and domestication grades. Genotypes with THC content > 0.3% are marked with *. Significant differences were found for both observed ASV richness and Shannon *H′* index (*P* ≤ 0.001, Kruskal–Wallis)

Alpha-diversity analysis, as measured by observed ASV richness and Shannon H′ index, was conducted based on a rarefied dataset (Fig. 1) Across the dataset, observed ASV richness ranged from 10 to 177 ASVs, with a median of 34 (*IQR* = 57–20.25) and Shannon H′ index from 0.14 to 2.58, with a median of 1.18 (*IQR* = 1.73–0.59) (Table S3). Significant differences in alpha diversity were found between genotypes and domestication grades for both measures (*P* ≤ 0.001, Kruskal–Wallis). Notably, Shannon H′ index values revealed a higher number of significant differences between genotypes, indicating uneven species abundances as a differentiating factor (Fig. S2). Regarding domestication, inbred lines exhibited the lowest diversity in both measures, followed by cross hybrids, whereas the highest diversity was observed in plants of low domestication grades, i.e. landraces and selected lines (Table S3). Differences in observed ASV richness between chemotypes were also significant (*P* ≤ 0.001) (Fig. S2). Differences in alpha diversity were still observed within the same provider (Table S4).

#### *Cannabis* seed microbiome structure is genotype specific, and line development reduces its dissimilarities

**Fig. 2:**
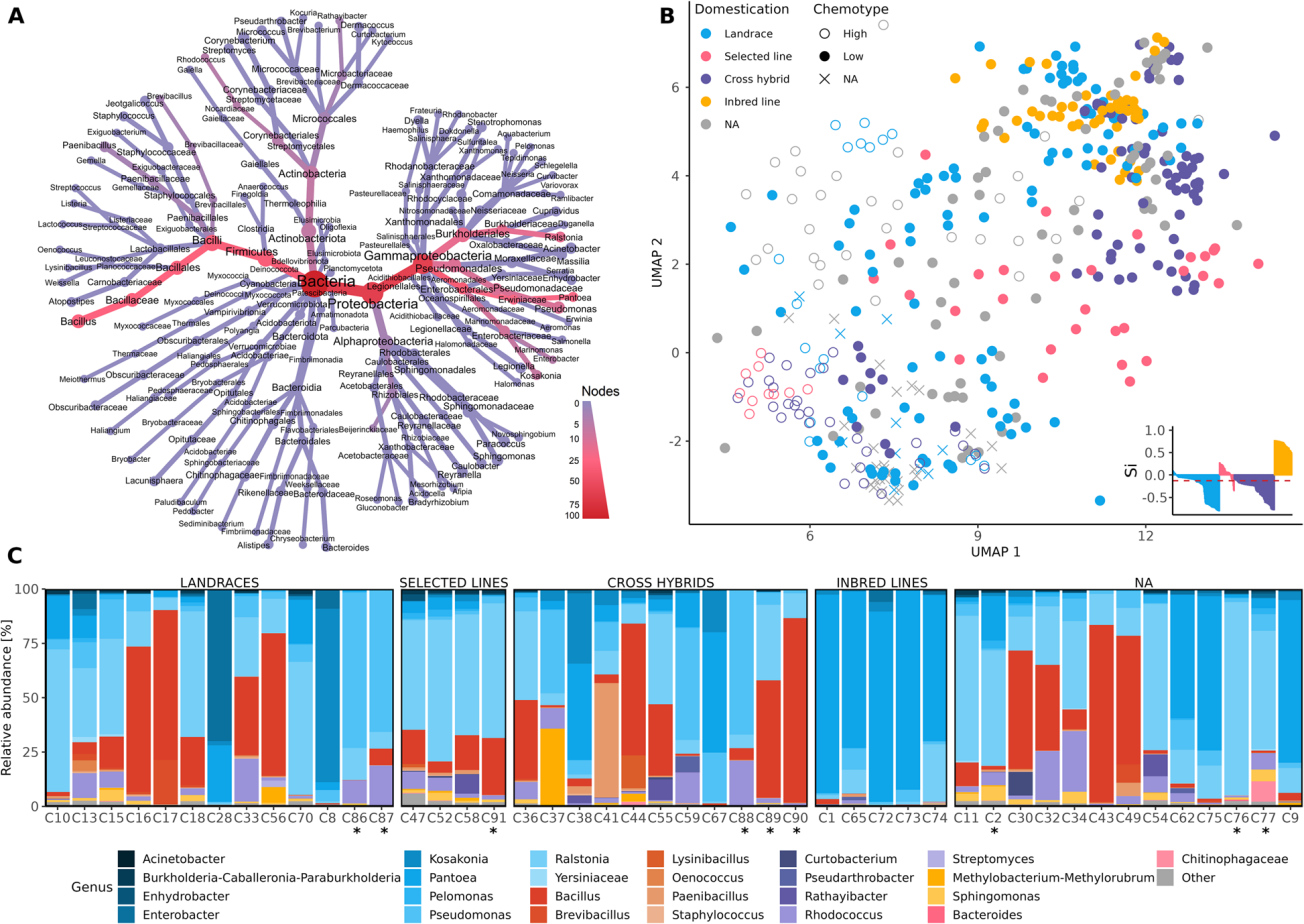
The structure of the *Cannabis* seed microbiome. **A** The heat tree shows the relative abundance (node colour) and the number of taxa (node size) of the identified seed endophytes on different taxonomic levels. **B** Dimensionality reduction of the amplicon sequencing variants (ASVs) using UMAP shows bacterial community composition based on a Bray–Curtis dissimilarity matrix, with significant differences between genotypes and domestication grades (*P* ≤ 0.001). The inset plot shows the separation distances between samples in the different domestication grades. Colours indicate different domestication grades and shape different chemotypes. **C** Mean relative abundance of the highly abundant (> 5%) members of the microbiome inhabiting seeds of different genotypes on genus level. Different genera are grouped according to bacterial classes using the same colour hue. Blue corresponds to Gammaproteobacteria, red to Bacilli, violet to Actinobacteria, yellow to Alphaproteobacteria, and pink to Bacteroidia, and the remaining genera are displayed in grey as *Other*. Genotypes with THC content > 0.3% are marked with *

The taxonomic composition of the *Cannabis* seed microbiome was investigated, revealing 813 different genera in 101 different classes belonging to 38 different phyla. Here, four predominant bacterial classes, Gammaproteobacteria, Bacilli, Actinobacteria and Alphaproteobacteria, collectively represented 99.49% of the reads in the dataset and 65.57% of ASV richness (Fig. 2A). *Bacillus* was identified as the most abundant genus with 22.09% of the reads, represented by 256 ASVs. This was followed by *Pantoea* with 54 ASVs covering 21.68% of the respective reads, while *Ralstonia* accounted for 137 ASVs, representing 21.64% of the reads. Other taxa with relative abundances above 1% were *Pseudomonas* (10.23%, 222 ASVs), *Rhodococcus* (5.04%, 65 ASVs), *Kosakonia* (4.47%, 14 ASVs), *Paenibacillus* (2.5%, 66 ASVs), *Enterobacter* (2.3%, 6 ASVs), *Methylobacterium-Methylorubrum* (1.83%, 44 ASVs), *Brevibacillus* (1.7%, 30 ASVs), *Rathayibacter* (1.36%, 51 ASVs) and *Sphingomonas* (1.07%, 82 ASVs).

To explore the influence of plant genotype and domestication grade on seed microbiome composition, ordination based on the Bray–Curtis dissimilarity matrix was conducted and visualized in a UMAP plot (Fig. 2B). Analysis of variance using adonis2 revealed significant differences in beta diversity among different genotypes (*P* ≤ 0.001), explaining 53.6% of the variations in bacterial composition. Pairwise comparisons demonstrated significant differences in bacterial community composition between the majority of the genotypes, with exceptions noted for specific pairs (Fig. S3). Furthermore, domestication explained 9.66% of the variations in bacterial composition (*P* ≤ 0.001). Differences in bacterial composition between chemotypes were also significant and explained 6.14% of the variations in bacterial composition (*P* ≤ 0.001). Moreover, significant differences in beta diversity among genotypes, domestication grades, and chemotypes within the same provider were observed (Table S4). Samples from genotypes associated with line development, i.e. selected lines and inbred lines, showed more positive silhouette coefficients, indicating a homogenization of their bacterial communities (Fig. 2b**)**.

Bacterial taxa with a relative abundance of at least 5% in the whole dataset were assessed for each genotype, resulting in the identification of 26 genera across five classes (Fig. 2C). Additionally, these were visualized for each sample across genotypes (Fig. S4). While all five classes were ubiquitous, their relative abundances varied. Gammaproteobacteria exhibited the highest diversity, dominating in 31 genotypes, followed by Bacilli in 13. Notably, certain genera were dominant in more plant genotypes, such as *Bacillus* and *Ralstonia* in 12 genotypes each and *Pantoea* and *Pseudomonas* in 11 and 5 genotypes, respectively. Actinobacteria predominantly occurred in one genotype (C54), primarily due to the genus *Rathayibacter*. The same was observed in C37 regarding Alphaproteobacteria, largely due to *Methylobacterium-Methylorubrum*. The class Bacteroidia did not emerge as dominant in any genotype, with its presence mainly observed in C77. The genotype C47 exhibited the highest percentage of reads outside these dominant classes and genera, albeit accounting for a minor proportion of the dataset.

#### Identification of bacterial markers linked to plant domestication

**Fig. 3:**
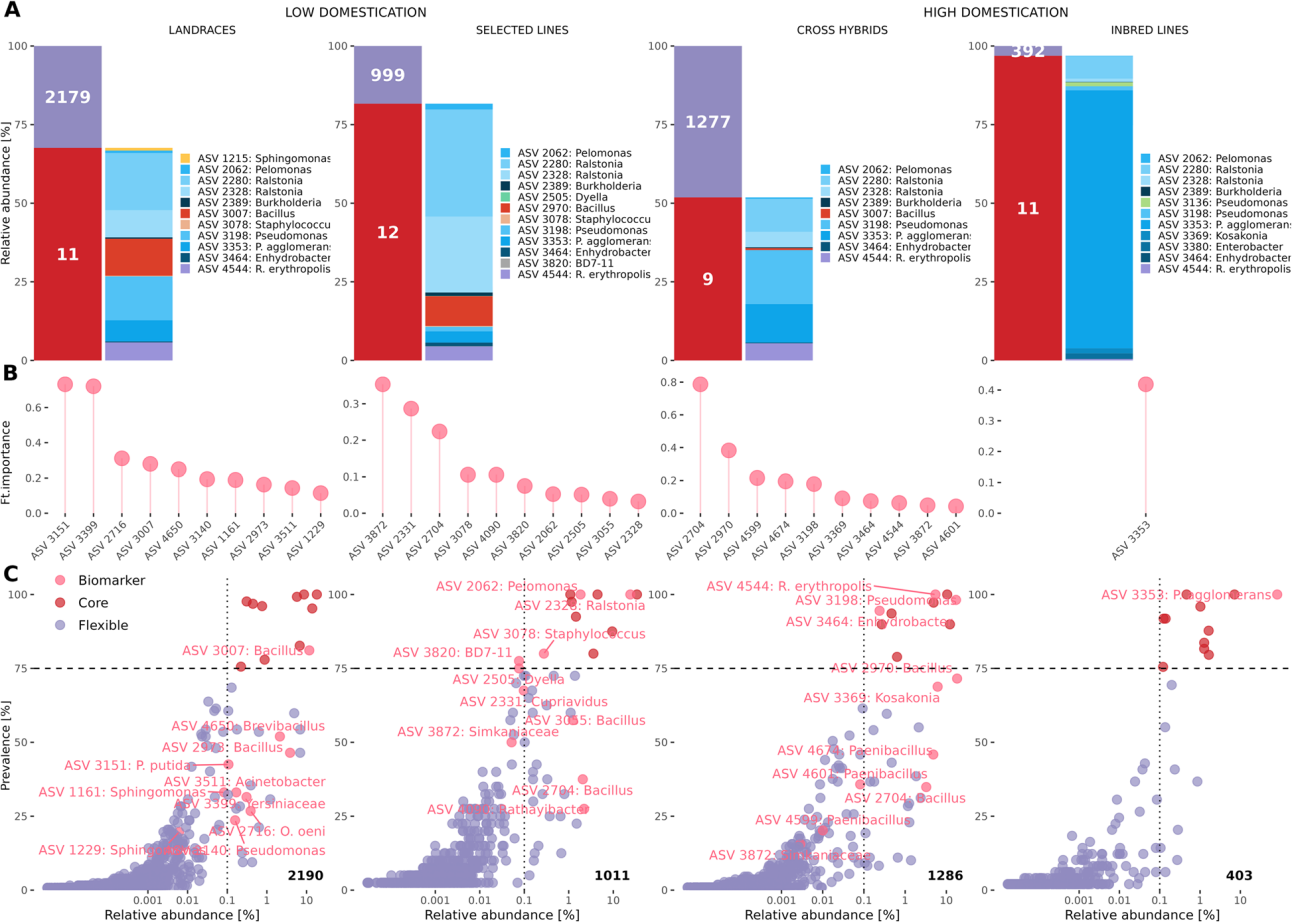
Abundance, taxonomy, and prevalence of core microbiome across domestication grades. **A** Cumulative relative abundance and richness of core (red) and flexible (violet) amplicon sequence variants (ASVs) and taxonomy of the core microbiome at the ASV level. **B** Top 10 ASVs (features, ft.) ranked according to their contribution to the gradient-boosted tree classification in the different domestication grades. Higher mean absolute SHAP values indicate higher importance for classification (*AUC* = 93.6% ± 0.0043%). **C** Abundance-occupancy curves showing the core (red) and flexible (violet) fractions of the microbiome as well as the identified biomarkers (pink)

We found that the seed core microbiome for each of the domestication grades representing ASVs shared between at least 75% of the samples within the group with a detection level of 0.1%. We found a similar number of core species within each domestication grade, however representing different cumulative relative abundances in each grade, whereas selection lines and inbred lines values reached up to 81.65% and 96.88%, respectively, while for landraces and cross hybrids core species represented 67.61% and 51.8%, respectively (Fig. 3A). In contrast, inbred lines are notably dominated by the presence of one ASV, presenting a relative abundance of 82.05%, i.e. *Pantoea agglomerans* (ASV 3353). Furthermore, 8 out of 17 core species found across grades, such as *Pelomonas* (ASV 2062), *Ralstonia* (ASV 2280 and ASV 2328), *Burkholderia *sp. (ASV 2389), *Pseudomonas *sp. (ASV 3198), *P.agglomerans* (ASV 3353), *Enhydrobacter* (ASV 3464) and *Rhodococcus erythropolis* (ASV 4544), were ubiquitous to all grades and collectively constitute a large fraction of the entire bacterial community with a cumulative relative abundance of 63.32% in the whole dataset (Fig. S5). Inbred lines and selected lines have shown to harbour more unique core members, including *Pseudomonas* (ASV 3136), *Kosakonia* (ASV 3369) and *Enterobacter* (ASV 3380) in inbred lines and *Bacillus* (ASV 2970), *Dyella* (ASV 2505) and *BD7-11* (ASV 3820) in selected lines. Landraces only have *Sphingomonas* (ASV 1215) as the unique core member, while cross hybrids have no unique core members.

The ML-based analysis with XGBoost revealed distinct microbial signatures associated with each domestication grade. ASVs such as *Pseudomonas putida* (ASV 3151), *Bacillus* (ASV 3007) and *Pseudomonas* (ASV 3140) were among the top 10 bacterial genera contributing to classification of landraces (Fig. 3B). Conversely, selection lines exhibited enrichment of taxa such as *Rathayibacter* (ASV 4090) and *Bacillus *sp. (ASV 3055). Cross hybrids included taxa such as *Bacillus* sp. (ASV 2704 and 2970) and *Paenibacillus* (ASV 4599, 4674, and 4601). Inbred lines had exclusively *P. agglomerans* (ASV 3353) as their signature, indicating a potential shift in the seed microbiome. These results underscore the potential of using seed microbiome profiles as biomarkers for plant domestication grades and provide insights into potentially beneficial taxa lost during domestication. The abundance and prevalence of the ASVs identified as biomarkers or core members for each domestication grade are presented in Fig. 3C.

#### Effects of the seed endophyte B. frigoritolerans C1141 on *Cannabis* growth and fitness

In light of the preceding findings, our investigation delved into the impact of the identified biomarkers from low domestication genotypes on *Cannabis* growth and fitness under both controlled and field conditions. From the obtained *Cannabis* seed isolates, a total of five biomarkers, three of landrace genotypes (ASV 3007: *Bacillus* sp., 3140: *Pseudomonas* sp., and 3151: *P.putida*) and two of selected lines (ASV 4090 *Rathayibacter* sp. and ASV 3055: *Bacillus* sp.), were identified with a 100% match in the V4 region and selected for further examination (Table S5).

**Fig. 4:**
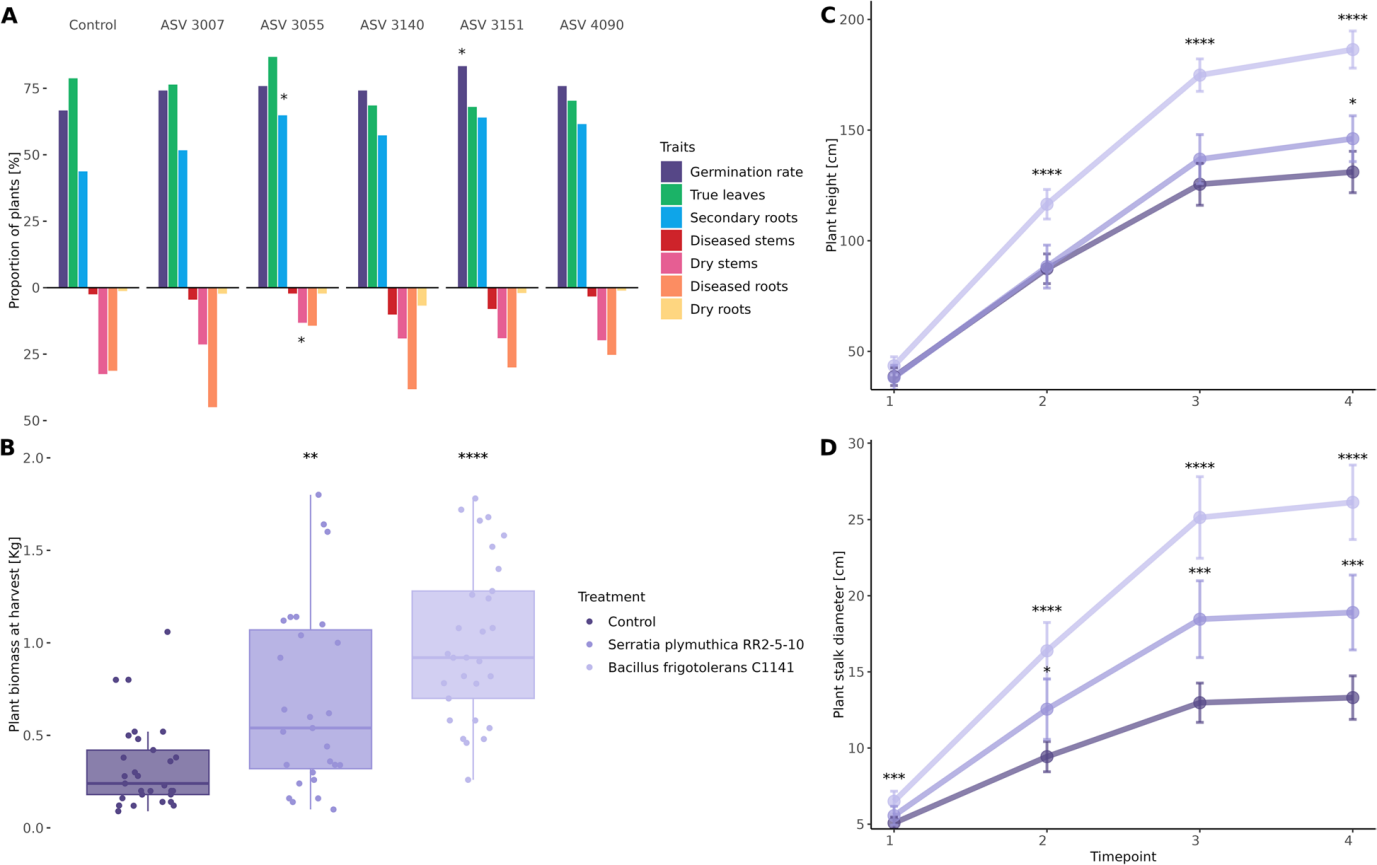
Influence of the different treatments tested *in planta* under controlled conditions (**A**) and in the field (**B**, **C**, **D**). **A** Comparison between the proportion of observed traits in *Cannabis* seedlings of the different treatments and the control under controlled conditions. Differences between treated and control *Cannabis* plants grown under field conditions on their **B** shoot fresh weight upon harvesting (*n* = 30 per group) and on their **C** heights and **D** stalk diameter at four different time points until the conclusion of the trial, whereas error bars represent the confidence interval of 95%. Significant differences are indicated as follows: * as *P* ≤ 0.05, ** as *P* ≤ 0.01, *** as *P* ≤ 0.001, and **** as *P* ≤ 0.0001

Priming of seeds with the aforementioned bacteria was implemented to assess potential effects on growth under controlled conditions. While all treatments, except for ASV 3140 (*Pseudomonas* sp.), exhibited enhancements in biomass, root and shoot length in 7-day-old seedlings, these improvements did not reach statistical significance (Fig. S6). Notably, treatment with ASV 3151 (*P.putida*) significantly increased the germination rate (*P* ≤ 0.05, *χ*^2^) (Fig. 4A), and the treatment with ASV 3055 (*Bacillus* sp.) emerged as the sole intervention to significantly improve secondary root formation (*P* ≤ 0.05, *χ*^2^) and mitigate the incidence of dead seedlings with dry stems. The latter was further identified as *B. frigotolerans*, assigned as strain C1141 and selected for further investigation under field conditions.

During field trials, the effects of *B. frigoritolerans* C1141 on *Cannabis* plants were compared with those of the patented plant-beneficial bacterium *S.plymuthica* RR2-5–10, as well as with untreated *Cannabis* plants. Results showed discernible differences in plant fresh weight upon harvest between the treatment groups and the control, with average yields 3 × higher in plants treated with *B. frigoritolerans* C1141 and 2 × higher in plants treated with *S.plymuthica* RR2-5–10 (Fig. 4B). Furthermore, treatment groups exhibited significant differences in plant height over the course of the trial, with marked improvements observed in plants treated with *B. frigoritolerans* C1141 from the second timepoint on and 43.3% higher at harvest, in contrast to those treated with *S.plymuthica* RR2-5–10, where an improvement in growth was only evident at the final timepoint (11.5%) (Fig. 4C). Remarkably, plants treated with *B. frigoritolerans* C1141 demonstrated a significant increase in stalk diameter as early as the first month (timepoint 1), a trend that persisted throughout the duration of the trial, reaching improvements of 96% at the time of harvest, which more than doubles the effects of *S.plymuthica* RR2-5–10 (42%) (Fig. 4D). These results provided a reliable confirmation of the benefits and adaptability of the seed microbiota when it comes to target approaches for the improvement of plant growth and fitness.

**Fig. 5:**
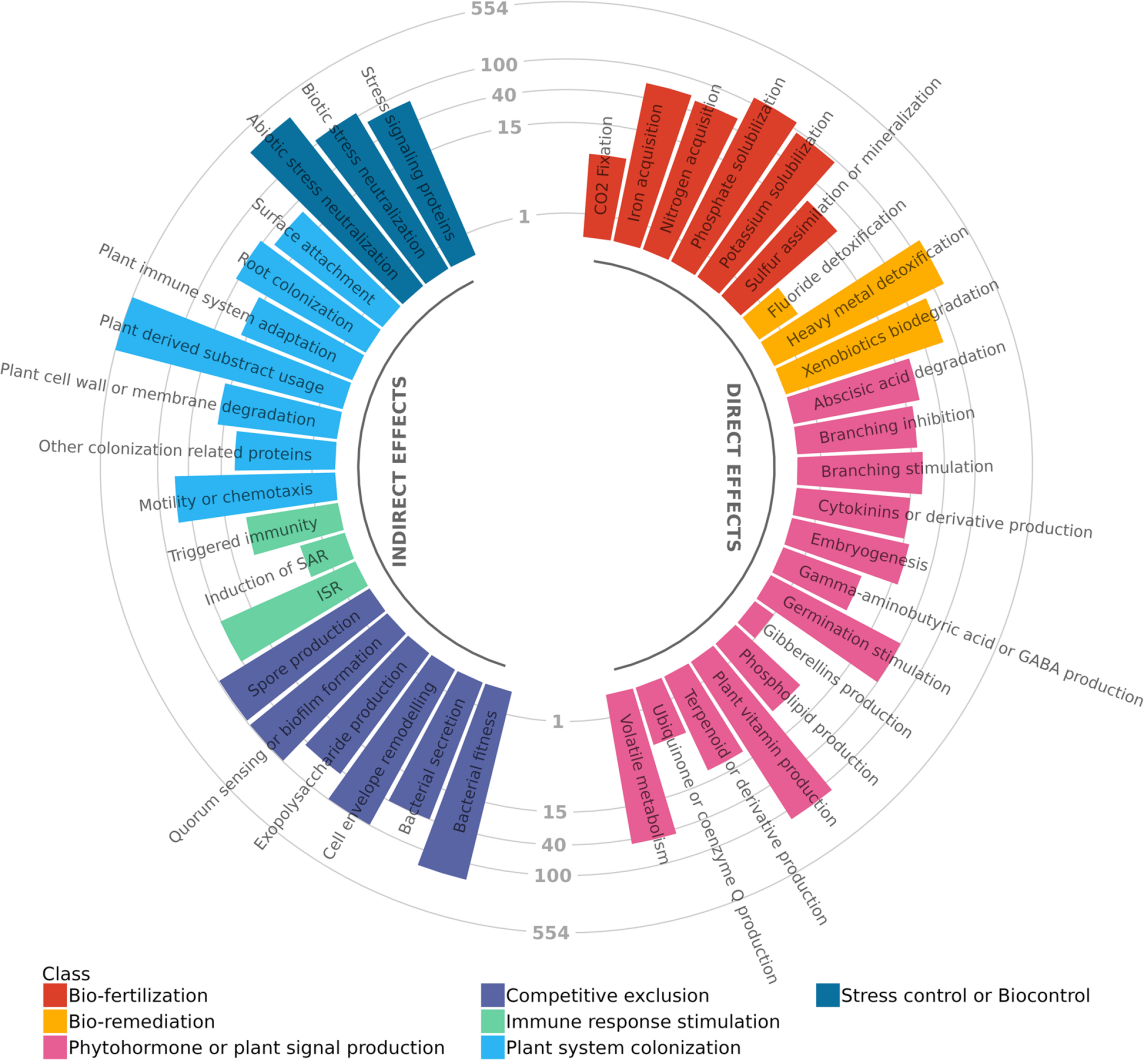
Predicted PGP functions of *B. frigoritolerans* C1141. Arches divide the identified PGP functions, represented by the bars, into direct and indirect effects. The height of each bar is shown in log_10_ scale and describes the number of genes found for each PGP function and the colours group the different PGP functions into different classes

Finally, we conducted a comprehensive analysis of strain C1141’s PGP functions using PLaBase. This approach offers a thorough characterization through genetic analysis, offering detailed insights into the multifaceted mechanisms underlying the potential of strain C1141 as an effective agent for promoting plant growth. A diverse array of 1572 distinct protein coding genes or gene clusters associated with various PGP functions was found and poised positively to impact plant growth, both directly (*n* = 591) and indirectly (*n* = 1315) (Fig. 5). Among the notable findings were numerous genes facilitating plant colonization (*n* = 678), particularly through

 the utilization of plant-derived substrates (*n* = 554) and mechanisms of motility or chemotaxis (*n* = 60), which are instrumental for seed-endophytic colonization via internal pathways. Additionally, a multitude of genes linked to phytohormone production (*n* = 254) were identified, with a notable abundance of genes involved in plant vitamin production (*n* = 132), volatile metabolism (*n* = 46), and germination stimulation (*n* = 40). A substantial number of genes associated with bio-fertilization *(n* = 263) were uncovered, primarily involved in phosphate (*n* = 120) and potassium (*n* = 93) solubilization, as well as iron (n = 62) and nitrogen (*n* = 55) acquisition. Moreover, our analysis revealed genes implicated in immune response stimulation (*n* = 51), particularly those involved in inducing systemic resistance (ISR) (*n* = 41), and in stress management or biocontrol (*n* = 445), aimed at mitigating both biotic (*n* = 111) and abiotic (*n* = 306) stress factors. Genes associated with competitive exclusion (*n* = 490), indicative of bacterial fitness (*n* = 162), and with mechanisms of quorum sensing or biofilm formation (*n* = 98) were identified, underscoring their importance in facilitating persistence within the plant ecosystem. Detailed descriptions of the top 25 gene features and associated genes are provided in Table S6.

## Discussion

In this work, we provide new insights to support harnessing the *Cannabis* seed microbiome for optimizing crop performance and sustainability in agricultural systems. The plant microbiome plays a pivotal role in shaping plant health and productivity. The process of domestication may have inadvertently disrupted the microbial equilibrium and symbiosis within currently produced plants. This could also include alterations in the microbiome composition and function that are fundamental for optimal growth and resilience against environmental stresses. Given the significant importance of seeds in ensuring the continuity of beneficial microbes, understanding the effects of domestication on the seed microbiome, particularly in economically significant crops like *Cannabis*, is paramount.

Here, we found evidence that the plant genotype is determinant in explaining the variation in the *Cannabis* seed bacterial diversity and composition. Although previous research has elucidated the pivotal role of the plant genotype in shaping the assembly of the seed microbiome [61], in *Cannabis*, it is a novel insight covering a highly heterogeneous genetic background. In analogy to other plant species, the diversity of *Cannabis* seed microbiome is lower than in other plant compartments [24, 25, 27]. The low moisture content and restricted nutrient availability inherent to seeds create an inhospitable environment for microbial endurance and proliferation [62]. Therefore, seeds are considered a bottleneck in the continuity of the plant microbiome. When compared to other plant species, *Cannabis* seeds harbour bacterial communities with low to intermediate diversity [63]. Here, differences in evenness of the bacterial communities were more determinant to distinguishing between genotypes than species richness. We have also found well-marked differences between the seed-endophytic community compositions of different *Cannabis* genotypes. These observations are in accordance with the strong effect of the plant genotype found in other above-ground compartments of *Cannabis* [24–27] and are supported by a smaller scale study with 16 genotypes [28]. Similar observations were being made in the seed-endophytic communities of other plant species [64–66]. The plant genotype generally shows a higher influence on these microbial communities than geographic location [67–70]. Although in the present study differences among plant genotypes from the same provider revealed selection by the host, pre- and postharvest variations, such as location, environmental fluctuations or even conditions and duration of storage, can still remain influential [71–73]. A detailed untangling of the interplay between host genetics and environmental effects on *Cannabis* seed microbiome assembly will require targeted experiments.

Domestication was also identified as a significant factor in shaping the seed-endophytic communities of *Cannabis*. Domestication, and breeding, wielded adeptly, can manipulate the genetic makeup of plants, leading to drastic changes in the seed microbiome, which has also been observed in other plants. Interestingly, the seed community structures differed between domestication grades, and genotypes originating from line development exhibited a higher clustering in comparison to landraces and cross hybrids. Hybridization between different genotypes can introduce novel genetic elements into the plant genome, potentially altering the interactions between the plant and its associated microbiota [74]. On the other hand, inbreeding involves repeated crosses within a limited gene pool, which can lead to the fixation of certain alleles and the loss of others through genetic drift [75]. Similarly, line development involves selecting and propagating individuals with specific traits over multiple generations, often within a narrow genetic background. While these breeding approaches aim to stabilize desired traits, they may inadvertently reduce genetic diversity and the potential for introducing novel genetic elements into the plant genome that could contribute to microbial diversity and interactions. In the present study, a significantly higher bacterial diversity was observed in low domestication grade genotypes (landraces and selected lines) in comparison to high domestication grade (cross hybrids and inbred lines), whereas selected lines showed the highest diversity. Studies have drawn diverging outcomes in this regard [76, 77], which reveal that the effects of domestication on the seed microbiome diversity are dependent on plant species.

In our study, differences between the bacterial composition of low and high THC genotypes were found. In *Cannabis*, breeding initiatives often coincide with the manipulation of cannabinoid ratios and potency and terpenoid profiles [78]. However, the impact of the interplay between breeding and secondary metabolites on the seed microbiota remains largely unexplored [79]. In a recent study, THC and THC acid were found to exhibit genotoxic effects towards certain bacteria, while other cannabinoids can show cytotoxic or oxidative effects [80]. It is likely that the accumulation of secondary metabolites within the trichomes of *Cannabis* flowers can act as a deterrent to pests and pathogens and thereby influence the microbial environment surrounding the developing seed.

Here, we used a broad range of *Cannabis* genotypes to comprehensively explore the bacterial community in *Cannabis* seed and the potential effects of different chemotypes. From the 101 different bacterial classes found, five — Gammaproteobacteria, Bacilli, Actinobacteria, Alphaproteobacteriaand Bacteroidia — were ubiquitous to all genotypes. Endophytic bacteria of the same phylogeny are commonly found in the seed endosphere of other plants [63]. In *Cannabis*, these bacteria have been previously reported in other plant compartments. For instance, bacteria belonging to Firmicutes and Proteobacteria — mostly *Bacillus* and *Pseudomonas* — were predominantly detected in the flower and leaf endospheres, while *Sphingomonas* and *Methylobacterium* have been previously found on their surfaces and Actinobacteria on the root endosphere [24, 26]. This can allude to recruitment from plant to seed via the internal and floral pathways [81]. Furthermore, these classes do not differ from the ones reported in hemp seeds [28]. However, genera like *Flavobacterium*, *Acidovorax*, *Herminiimonas*, *Chryseobacterium* and *Massilia*, therein described as most abundant, compose members of the rare taxa in our study, with relative abundances similar or less than 0.01%. Moreover, neither *Flavobacterium* nor *Herminiimonas* are highly prevalent among the analysed genotypes in our study. Such differences could have arisen from the harsh sterilization method applied by the authors. A previous study focusing on the surface sterilization effects on endophytes in tea leaves and stems indicated that *Bacillus* has the highest susceptibility to high concentrations of NaClO [82]. The extent to which these treatments can affect certain types of taxa and yield false negatives can be dependent on their exact localization inside the seed tissues.

The spanning diversity of *Cannabis* genotypes tested, covering great part of its versatility and the polymorphic amplitude, allowed to refine the identification of shared ASVs. We found that both selected and inbred lines harboured more unique core members in comparison to the other domestication grades. Herein, ASVs shared within the same domestication grade and can point to potentially important *M* gene-bacteria associations that were affected by the domestication process. *M* genes, short for microbiome genes, were recently introduced to classify plant genes that are linked to the microbiome [83]. In addition, generally shared ASVs, ubiquitous to all domestication grades, can provide evidence of evolutionary conservation independently of domestication and chemotype. Core genera like *Pantoea*, *Pseudomonas*, *Ralstonia* and *Burkholderia* seem to be widespread across seeds of different plant species, which may point to important physiological functions in spermatophytes [63]. From the core microbiome of low to high domestication grades, we observed an enrichment of members of the order Enterobacteriales, i.e. *Kosakonia* and *Enterobacter* and *Pantoea*, in the core microbiome of inbred lines and a reduction/depletion of Bacillales, i.e. *Staphylococcus* and *Bacillus*. Notably, a large proportion of *P. agglomerans* (ASV 3353) was found in the core of inbred lines. *P. agglomerans* has known plant growth promoting [84, 85], as well as antagonistic properties towards phytopathogens [86], and can be vertically transmitted across plant generations [87]. Previous research has consistently detected a higher abundance of Bacteroidetes in the root-associated microbiome of wild relatives, while an enrichment of Actinobacteria and Proteobacteria was observed with modern crops [88, 89]. These observations might convey the influence of soil management practices and are not fully translated to the microbiome of seeds. In seeds, the influence of domestication on the microbial communities has been reported to be dependent on the resulting plant phenotype [90], and an enrichment in *Pantoea* has been associated with intensive breeding in other species [76, 77, 91].

While it is not clear what consequences this enrichment might have in terms of plant fitness, it would be interesting to address which *M* genes might be responsible for it.

Based on the diversity assessments performed in this study, we decided to search for particular ASVs that may support growth or fitness of *Cannabis* plants but were lost during the domestication process, as suggested by Raaijmakers and Kiers [92]. Hence, we consider shifts in the seed microbiome not only a reflection of the domestication state of the plant but also responsible for plant fitness. The present study used rather short 16S rRNA gene fragments to confirm sequence matches between amplicon data and sequences obtained from isolates. Future approaches would profit from full-length 16S rRNA sequencing of amplicons from seed material for more precise identifications.

The field trial results conducted in this study confirmed the capability of the selected isolate *B. frigoritolerans* C1141 to promote growth in *Cannabis* cuttings from a cross hybrid. Most notably, this bacterium outperformed the beneficial effects of the already established plant beneficial *S. plymuthica* RR2-5–10, isolated from the rhizosphere of oilseed rape. These results not only supported seeds, as a useful source of beneficial microorganisms, but also that the modulation of the seed microbiome through personalized interventions may help to restore plant homeostasis and improve plant health. Moreover, these results also apprise to the importance of under-domesticated genotypes like locally adapted landraces, which can provide a repository to reintroduce microbial diversity into domesticated populations. This is especially true in *Cannabis* whose natural seed microbial transmission got interrupted by phytosanitary requirements or vegetative propagation practices [93]. Furthermore, the comprehensive characterization of *B. frigoritolerans* C1141 genomic features provided important insights in our understanding of its functional capabilities. This strain harboured a considerable number of genes and gene clusters related to plant colonization, phytohormone production, bio-fertilization, immune response stimulation, stress control and competitive exclusion. These genetic features can explain not only its ability to successfully colonize *Cannabis* plants and eventually the seeds but also the observed beneficial outcomes in *Cannabis*.

## Conclusion

Our study provides new insights related to the impact of plant genotype, domestication and breeding on endophytic bacterial communities in *Cannabis* seeds. *Cannabis* genotypes shared a fraction of predominant bacteria but presented significant differences in terms of diversity and composition. Our data showed further microbial shifts related to domestication and breeding in terms of composition, diversity and abundance. Within the frame of the rewilding hypothesis, we were able to promote *Cannabis* fitness when reinstating a bacterial seed endophyte associated with low domestication. This work highlights the potential of leveraging seed-associated microorganisms to enhance plant growth and resilience and the importance of considering microbiome-assisted crop improvement or fine-tuning seed endophyte composition via *M* gene breeding for sustainable *Cannabis* production by reducing the need for chemical inputs.

## Supplementary Information

Supplementary Material 1: Fig. S1 Alpha rarefaction at 5607 reads per sample represented across (a) genotypes, (b) domestication grades and (c) chemotypes. Fig. S2 Pairwise comparison in (a) ASV-based observed richness and (b) Shannon H’ diversity for the different genotypes. Pairwise comparison in (c) ASV-based observed richness and (d) Shannon H’ diversity for the different domestication grades. Pairwise comparison in (e) ASVbased observed richness and (f) Shannon H’ diversity for the different chemotypes. Significant differences (α = 0.05) are marked (*). Fig. S3 Pairwise comparison of the Bray-Curtis dissimilarity matrix between (a) *Cannabis* genotypes, (b) domestication grades and (c) chemotypes. Significant differences (α = 0.05) are marked (*). Fig. S4 Bacterial composition at genus level in replicate samples across genotypes (detection level >0.05). Fig. S5 Abundance, taxonomy, and prevalence of core microbiome across the whole dataset represented by ASVs shared between at least 75% of the samples between domestication grades with a detection level of 0.1%. (a) Abundance-occupancy curves showing the core (red) and flexible (violet) fractions of the microbiome. (b) Cumulative relative abundance and richness of core (red) and flexible (violet) amplicon sequencing variants (ASVs) and (c) taxonomy of the core microbiome at the ASV level. Fig. S6 Influence of the different treatments in planta under controlled conditions regarding plant length (a) and biomass (b). Error bars represent the 95% confidence interval. Table S1 Detailed information on the *Cannabis* genotypes used in this study. LC refers to THC content <0.3%. Table S2 Hyperparameters of gradient boosted-trees. Table S3 Statistics of the calculated alpha diversity indices described by median and interquartile range (IQR) for each *Cannabis* (a) genotype, (b) domestication grade and (c) chemotype in the rarefied dataset. Table S4 Permutational multivariate analysis (permanova, 999 permutations) with adonis2 in Vegan and Kruskal-Wallis rank sum test on *Cannabis* genotype, domestication grade and chemotype within the same provider to assess differences in beta and alpha diversity, respectively. The last row shows the differences between providers. Significant differences (α = 0.05) are marked (*). Table S5 Identity and alignment report between identified biomarker ASVs and isolated bacteria from *Cannabis* seed endosphere. Table S6 Top 25 gene features found in* Bacillus frigotolerans* C1141 genome, regarding the number of associated genes.

## Data Availability

The assembled *Bacillus frigotolerans* C1141 genome, along with the associated annotations, was deposited in the National Center for Biotechnology Information (NCBI; https://www.ncbi.nlm.nih.gov/) under accession number PRJNA1113337. The 16S rRNA gene amplicon dataset was deposited in the European Nucleotide Archive (ENA; https://www.ebi.ac.uk/ena) under the accession number PRJEB64469. The bioinformatic and statistical analysis pipeline and files for reproducibility are available in the GitHub repository, https://github.com/cbclobato/wild-again.

## Abbreviations

ASVs: Amplicon sequencing variants
AUC: Area under the receiver operating curve
CBD: Cannabidiol
CLR: Continuous long-read sequencing
CSS: Cumulative sum scaling
IQR: Interquartile range
ML: Machine learning
NaClO: Sodium hypochlorite
NA II: Nutrient agar II
NB II: Nutrient broth II
OD_600_: Optical density at a wavelength of 600 nm
PGP: Plant growth promoting
PNA: Peptide nucleic acid
SHAP: SHapley Additive exPlanations
THC: Tetrahydrocannabinol
WGS: Whole genome sequencing
