# Supplementary material for: Wild again: recovery of a beneficial Cannabis seed endophyte from low domestication genotypes
Source: Microbiome. 2024 Nov 15;12:239. doi: 10.1186/s40168-024-01951-5 (PMC11568533; doi:10.1186/s40168-024-01951-5)
Supplement: Supplementary file 2 — Supplementary Material 1: Fig. S1 Alpha rarefaction at 5607 reads per sample represented across (a) genotypes, (b) domestication grades and (c) chemotypes. Fig. S2 Pairwise comparison in (a) ASV-based observed richness and (b) Shannon H’ diversity for the different genotypes. Pairwise comparison in (c) ASV-based observed richness and (d) Shannon H’ diversity for the different domestication grades. Pairwise comparison in (e) ASVbased observed richness and (f) Shannon H’ diversity for the different chemotypes. Significant differences (α = 0.05) are marked (*). Fig. S3 Pairwise comparison of the Bray-Curtis dissimilarity matrix between (a) Cannabis genotypes, (b) domestication grades and (c) chemotypes. Significant differences (α = 0.05) are marked (*). Fig. S4 Bacterial composition at genus level in replicate samples across genotypes (detection level >0.05). Fig. S5 Abundance, taxonomy, and prevalence of core microbiome across the whole dataset represented by ASVs shared between at least 75% of the samples between domestication grades with a detection level of 0.1%. (a) Abundance-occupancy curves showing the core (red) and flexible (violet) fractions of the microbiome. (b) Cumulative relative abundance and richness of core (red) and flexible (violet) amplicon sequencing variants (ASVs) and (c) taxonomy of the core microbiome at the ASV level. Fig. S6 Influence of the different treatments in planta under controlled conditions regarding plant length (a) and biomass (b). Error bars represent the 95% confidence interval. Table S1 Detailed information on the Cannabis genotypes used in this study. LC refers to THC content <0.3%. Table S2 Hyperparameters of gradient boosted-trees. Table S3 Statistics of the calculated alpha diversity indices described by median and interquartile range (IQR) for each Cannabis (a) genotype, (b) domestication grade and (c) chemotype in the rarefied dataset. Table S4 Permutational multivariate analysis (permanova, 999 permutations) with adonis2 [file 40168_2024_1951_MOESM1_ESM.docx]

Supporting Information

**Wild again: Recovery of a beneficial *Cannabis* seed endophyte from low domestication genotypes**

Carolina Lobato^1^, João Machado de Freitas^2^, Daniel Habich^1^, Gabriele Berg^1,3,4^, Tomislav Cernava^1,5^

^1^ Institute of Environmental Biotechnology, Graz University of Technology, Petersgasse 12, 8010 Graz, Austria.

^2^ Signal Processing and Speech Communication Laboratory, Graz University of Technology, Inffeldgasse 16c/EG, 8010 Graz, Austria

^3^ Leibniz Institute for Agricultural Engineering and Bioeconomy, Max-Eyth-Allee 100, 1446 Potsdam, Germany.

^4^ Institute for Biochemistry and Biology, University of Potsdam, Karl-Liebknecht-Str. 24-25, 14476 Potsdam OT Golm, Germany

^5^ School of Biological Sciences, Faculty of Environmental and Life Sciences, Highfield Campus, SO17 1BJ Southampton, UK.

* Corresponding author: tomislav.cernava@tugraz.at | +43 316 873 8312


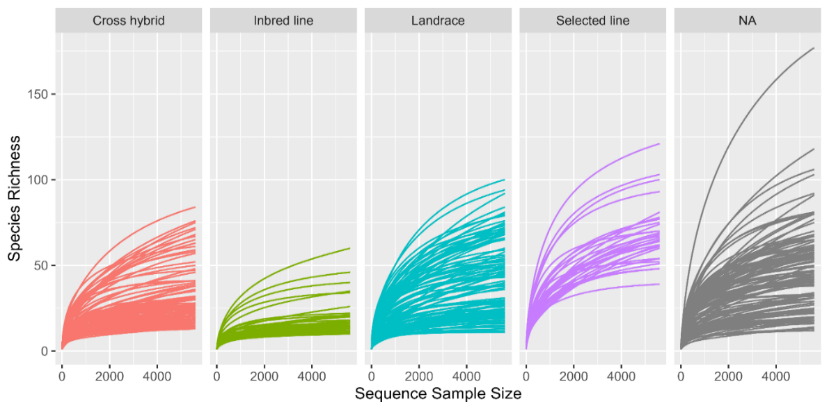


**b**


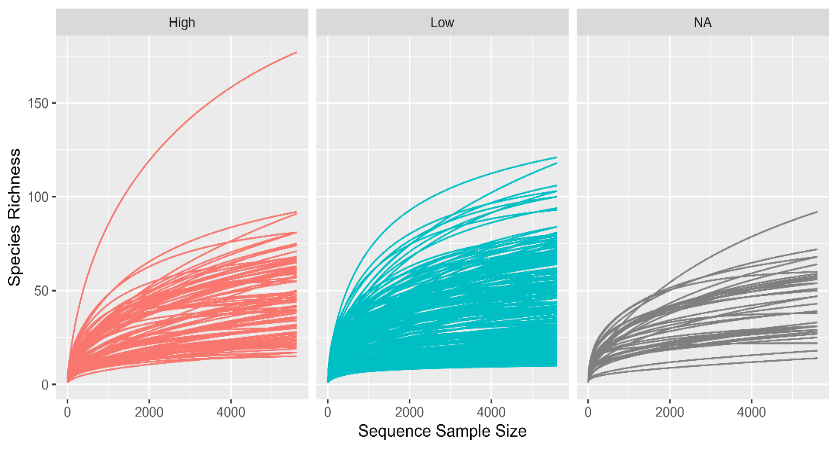


**c**


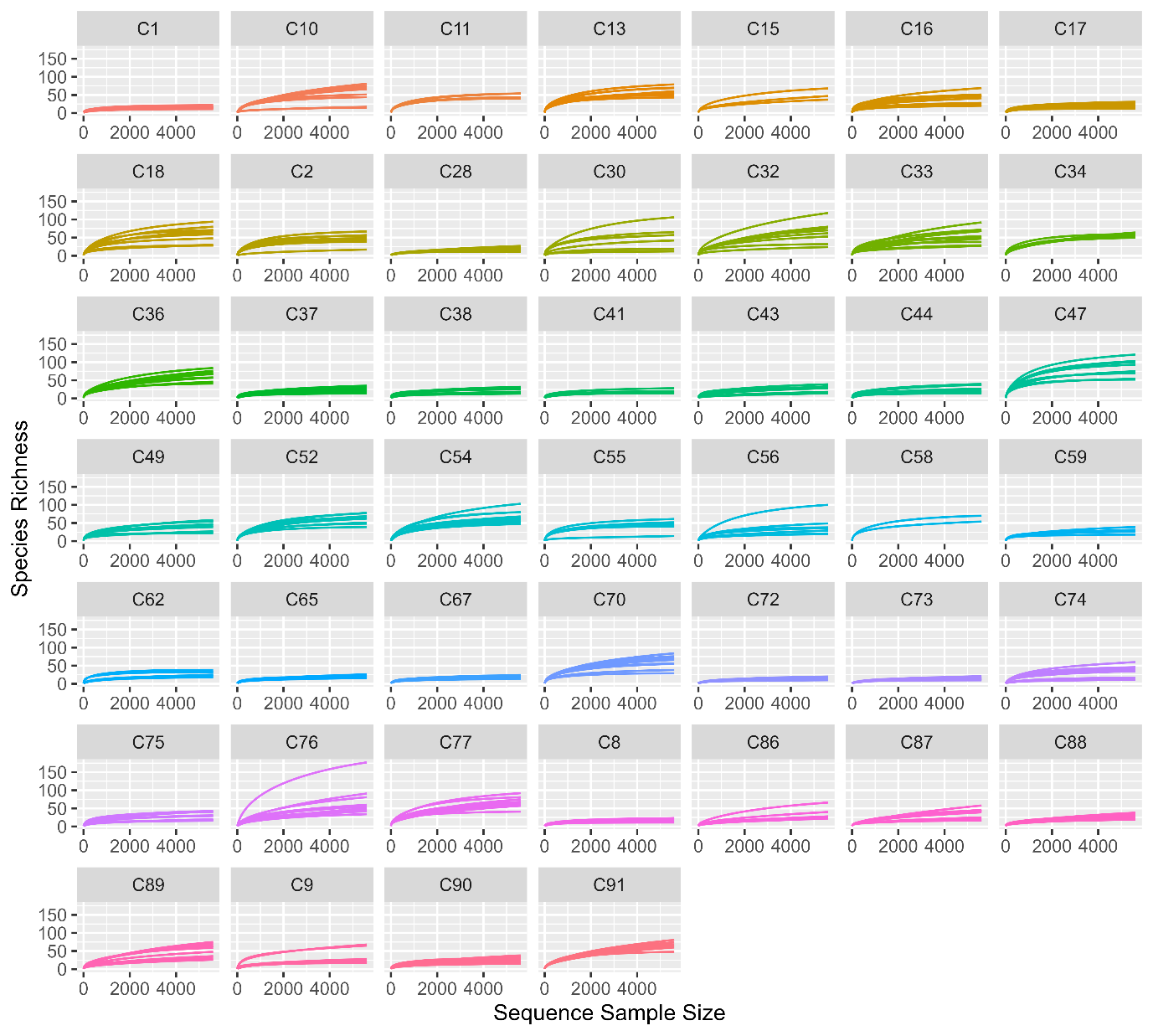


**a**

**Fig. S1** Alpha rarefaction at 5607 reads per sample represented across **(a)** genotypes, **(b)** domestication grades and **(c)** chemotypes.


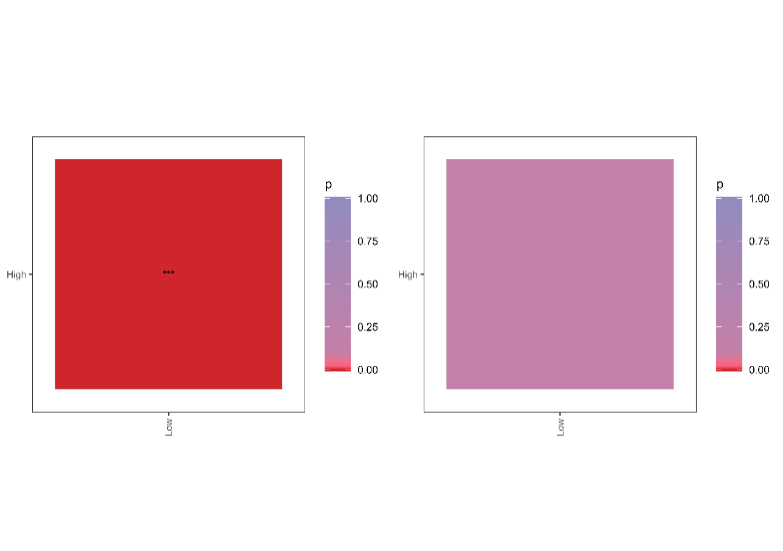


**e**

**f**


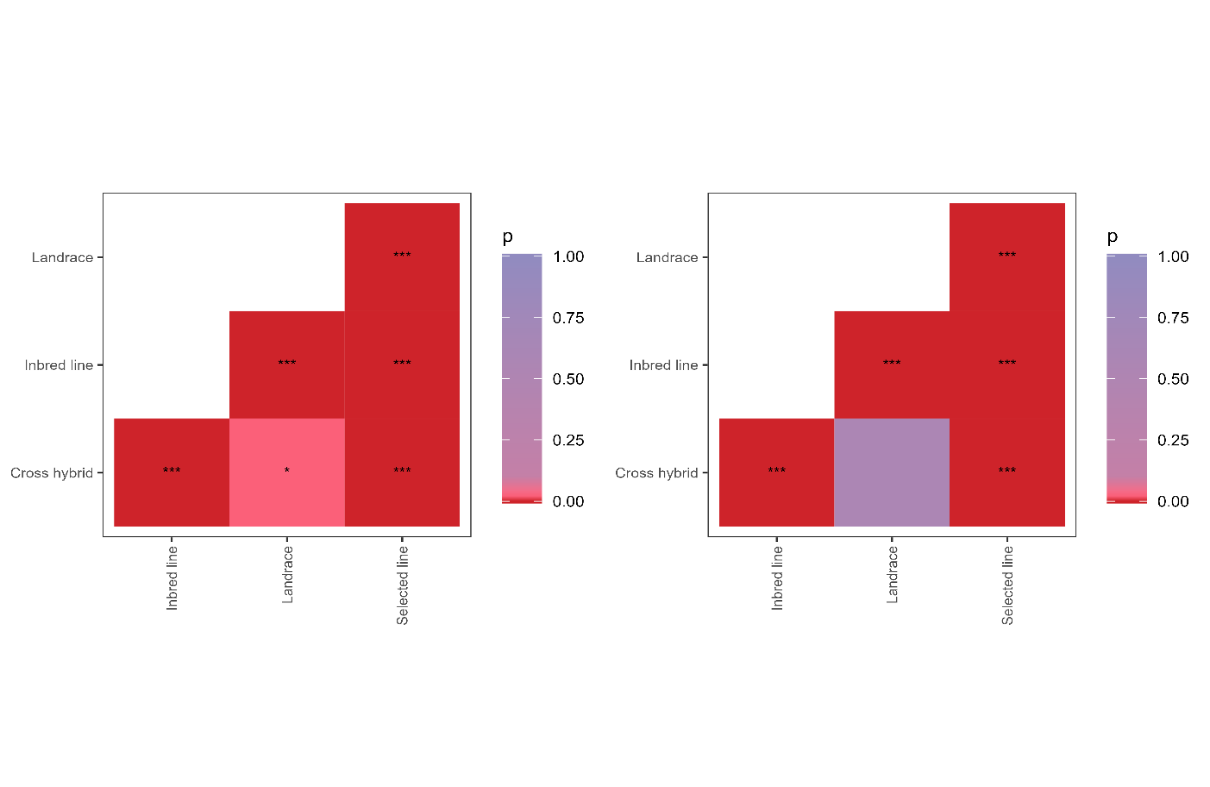


**c**

**d**


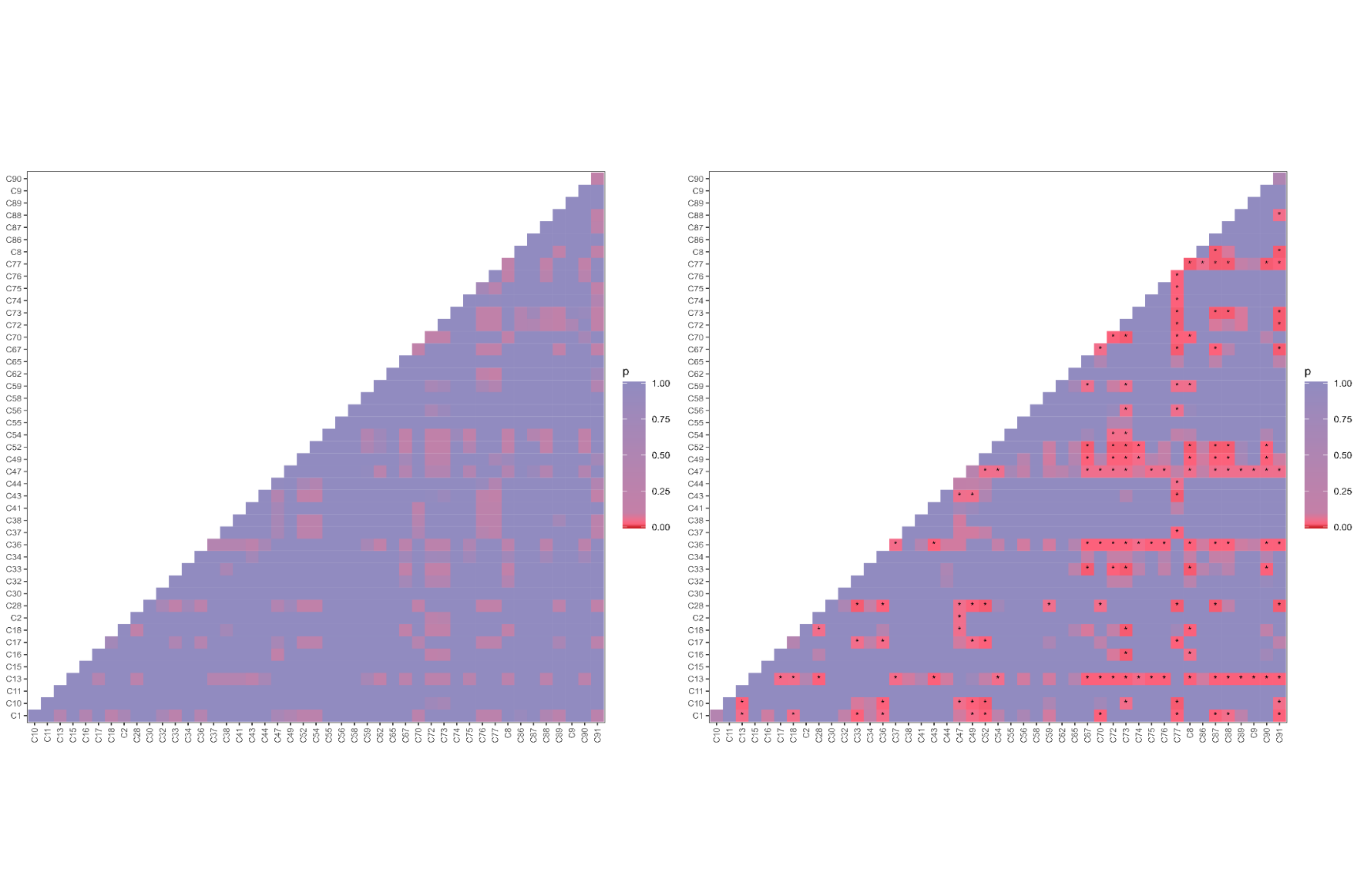


**a**

**b**

**Fig. S2** Pairwise comparison in **(a)** ASV-based observed richness and **(b)** Shannon H’ diversity for the different genotypes. Pairwise comparison in **(c)** ASV-based observed richness and **(d)** Shannon H’ diversity for the different domestication grades. Pairwise comparison in **(e)** ASV-based observed richness and **(f)** Shannon H’ diversity for the different chemotypes. Significant differences (α = 0.05) are marked (*).

**c**

**b**

**a**


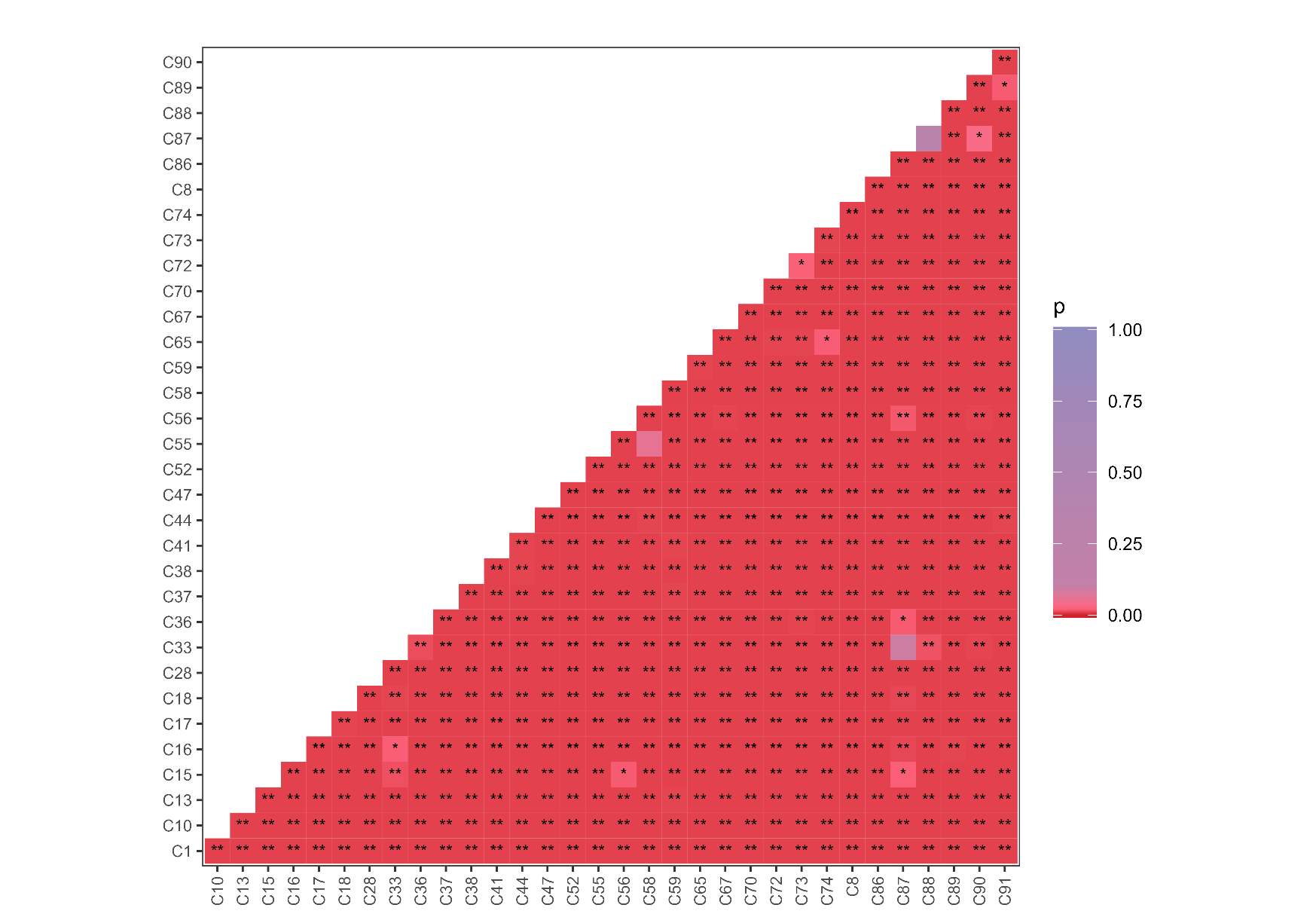

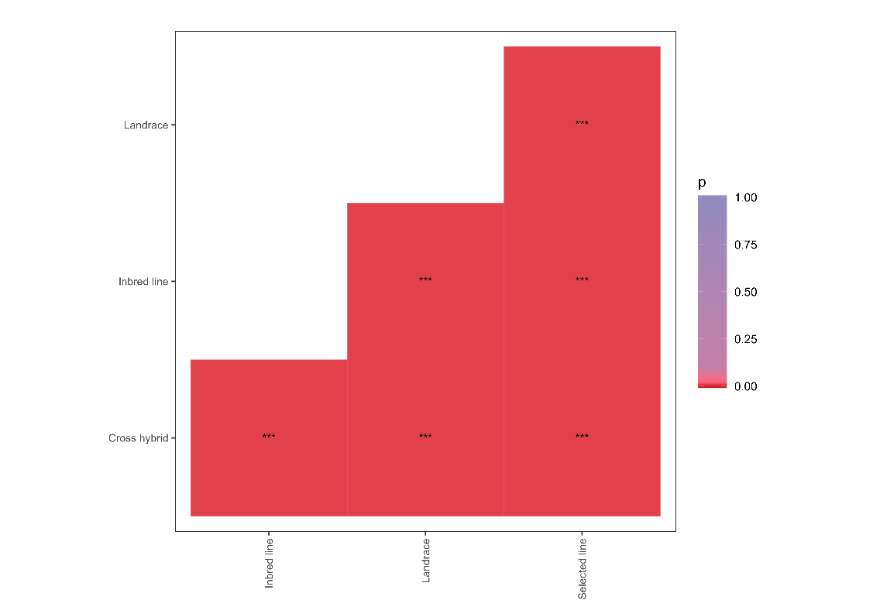

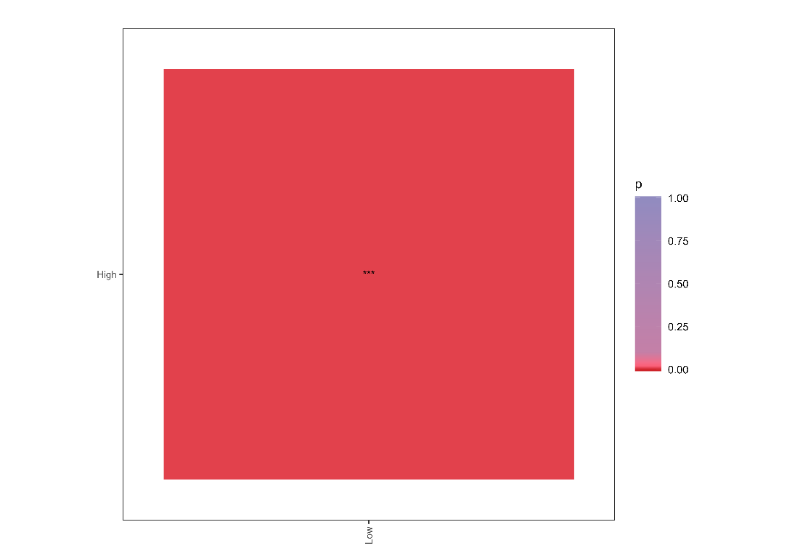


**Fig. S3** Pairwise comparison of the Bray-Curtis dissimilarity matrix between **(a)** *Cannabis* genotypes, **(b)** domestication grades and **(c)** chemotypes. Significant differences (α = 0.05) are marked (*).


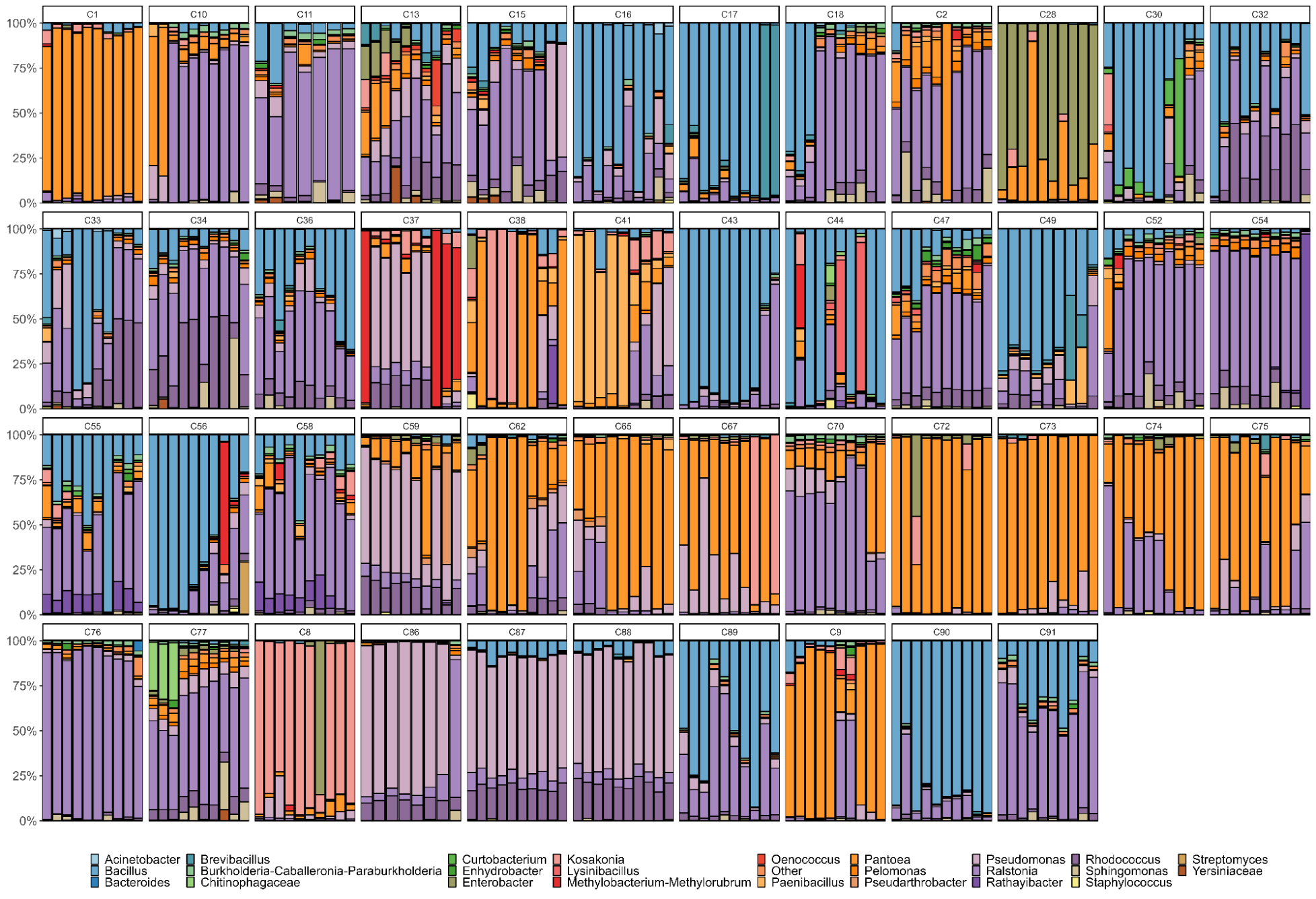


**Fig. S4** Bacterial composition at genus level in replicate samples across genotypes (detection level >0.05).


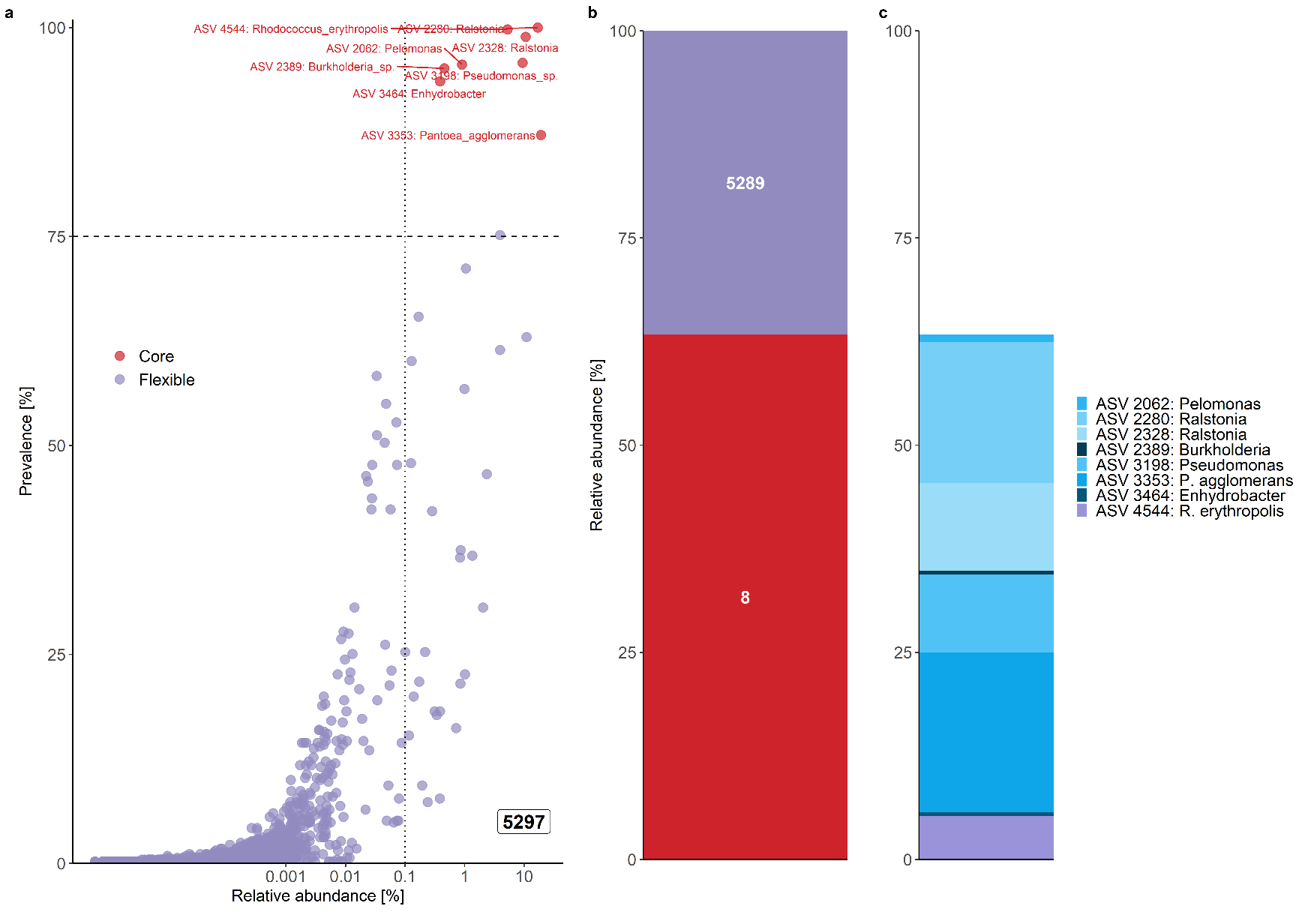


**Fig. S5** Abundance, taxonomy, and prevalence of core microbiome across the whole dataset represented by ASVs shared between at least 75% of the samples between domestication grades with a detection level of 0.1%. **(a)** Abundance-occupancy curves showing the core (red) and flexible (violet) fractions of the microbiome. **(b)** Cumulative relative abundance and richness of core (red) and flexible (violet) amplicon sequencing variants (ASVs) and **(c)** taxonomy of the core microbiome at the ASV level.


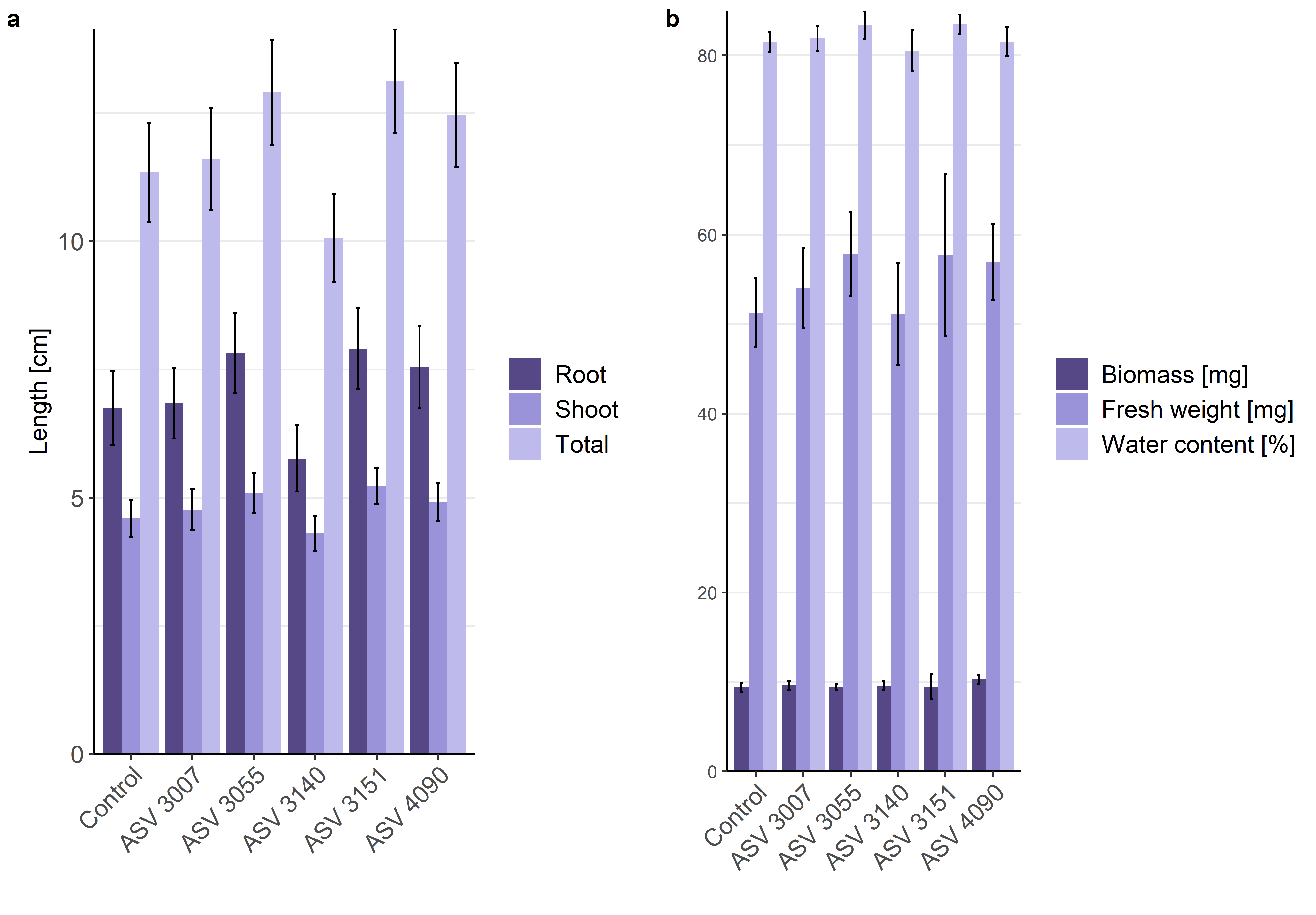


**Fig. S6** Influence of the different treatments in planta under controlled conditions regarding plant length **(a)** and biomass **(b)**. Error bars represent the 95% confidence interval.

**Table S1** Detailed information on the *Cannabis* genotypes used in this study. LC refers to THC content <0.3%.

| **Provider** | **ID** | **Genotype** | **Origin** | **Accession** | **Species** | **Domestication grade** | **Pedigree** | **Chemotype** | **Reproductive**  **type** | **References** |
| --- | --- | --- | --- | --- | --- | --- | --- | --- | --- | --- |
| CRI | C65 | Beniko | Poland | 1500003 | *C. sativa ssp. sativa var. sativa* | Inbred line | Fibrimon 24 x Fibrimon 21 | LC | Monoecious | [1], [2], [3], [4], [5] |
|  | C67 | Finola | Finland | 1500019 | *C. sativa ssp. sativa var. sativa* | Cross hybrid | NA | LC | Dioecious | [6], [7], [8], [9] |
| Green House Seeds Co. | C77 | Arjan's Haze #3 | NA | GHSC052 | *C. sativa ssp. indica var. indica* | Cross hybrid S1 | (Mexican x Colombian x Thai x Indian) x Laos landrace | THC- enriched | Dioecious | [10] |
|  | C76 | The Church CBD | NA | GHSC005 | *C. sativa ssp. indica var. indica* | Cross hybrid S1 | Afghani 1 x Mexican Sativa x Skunk 1 | CBD/THC  - enriched | Dioecious | [11] |
| IPK | C54 | Bredemann P | Germany | CAN 62 | *C. sativa ssp. sativa* | NA | NA | LC | NA | [5], [12], [13] |
|  | C28 | Cinepa | Bulzestii de Sus, Romania | CAN 35 | *C. sativa ssp. sativa* | Landrace | NA | LC | NA | [4], [14] |
|  | C41 | Eletta Campana | Italy | CAN 48 | *C. sativa ssp. sativa var. sativa* | Cross hybrid | Carmagnola x Fibridia | LC | Dioecious | [5], [15], [16], [17] |
|  | C59 | Fasamo | Germany | CAN 67 | *C. sativa ssp. sativa var. sativa* | Cross hybrid | Schurig x Bernburger-Einhausigen | LC | Monoecious | [5], [18], [19] |
|  | C44 | Fibridia | Germany | CAN 52 | *C. sativa ssp. sativa var. sativa* | Cross hybrid | Havelländer x Italian and Turkish landraces | LC | Dioecious | [5], [20], [21] |
|  | C37 | Fibrimon | Germany | CAN 44 | *C. sativa ssp. sativa var. sativa* | Cross hybrid | Havelländer x Schurig x Italian and Turkish landraces | LC | Monoecious | [5], [15], [21], [22] |
|  | C47 | Havelländer | Germany | CAN 55 | *C. sativa ssp. sativa var. sativa* | Selected line | Russian landraces | LC | Dioecious | [5], [23] |
|  | C36 | Hohenthürmer Gleichzeitig Reifender | Germany | CAN 43 | *C. sativa ssp. sativa var. sativa* | Cross hybrid | Waag valley landraces x Schurig x Kuhnow | LC | Subdioecious | [24], [25] |
|  | C62 | Kompolti | Hungary | CAN 70 | *C. sativa ssp. sativa var. sativa* | NA | Fleischmann hemp | LC | Dioecious | [5], [15], [17], [26] |
|  | C38 | Krasnoyarskaya | Russia | CAN 45 | *C. sativa ssp. sativa var. sativa* | Cross hybrid | Russian landraces x Italian landraces | LC | Dioecious | [5], [15], [17], [27] |
|  | C58 | Lovrin 110 | Romania | CAN 66 | *C. sativa ssp. sativa var. sativa* | Selected line | Bulgarian Silistra landraces | LC | Dioecious | [5], [17], [28], [29] |
|  | C43 | NA Argentina | Argentina | CAN 51 | *C. sativa ssp. sativa* | NA | NA | NA | NA | [30] |
|  | C34 | NA BG Belgium | NA | CAN 41 | *C. sativa ssp.* | NA | NA | NA | NA | [31] |
|  | C32 | NA BG China | China | CAN 39 | *C. sativa ssp. sativa* | NA | NA | LC | NA | [4], [32] |
|  | C11 | NA BG Hungary | Hungary | CAN 17 | *C. sativa ssp. sativa* | NA | NA | LC | Dioecious | [4], [15], [33] |
|  | C33 | NA BG Italy | Carmagnola, Italy | CAN 40 | *C. sativa ssp.* | Landrace | NA | NA | NA | [34] |
|  | C15 | NA BG Romania | Laşi, Romania | CAN 21 | *C. sativa ssp. sativa* | Landrace | NA | LC | Dioecious | [4], [15], [35] |
|  | C30 | NA France | France | CAN 37 | *C. sativa ssp. sativa* | NA | NA | LC | NA | [4], [36] |
|  | C49 | NA Syria | Syria | CAN 57 | *C. sativa ssp.* | NA | NA | NA | NA | [37] |
|  | C56 | NA Turkey | Burdur, Turkey | CAN 64 | *C. sativa ssp. sativa var. sativa* | Landrace | NA | LC | Dioecious | [15], [38] |
|  | C55 | Ramo | Germany | CAN 63 | *C. sativa ssp. sativa var. sativa* | Cross hybrid | NA | LC | Dioecious | [25], [39] |
|  | C16 | RV Georgia 86 | Shatili, Georgia | CAN 22 | *C. sativa ssp. sativa var. spontanea* | Landrace | NA | LC | Dioecious | [4], [15], [40] |
|  | C18 | RV Italy 84 | Apúlia, Italy | CAN 24 | *C. sativa ssp. sativa* | Landrace | NA | LC | Dioecious | [4], [15], [41] |
|  | C13 | RV Italy 88 | Cosenza, Italy | CAN 19 | *C. sativa ssp. sativa* | Landrace | NA | LC | Subdioecious | [4], [15], [42] |
|  | C17 | RV Korea 87 | North Hwanghae, North Korea | CAN 23 | *C. sativa ssp. sativa var. sativa* | Landrace | NA | LC | Dioecious | [4], [15], [43] |
|  | C10 | RV Slovakia 77 | Uličské Krivé, Slovakia | CAN 16 | *C. sativa ssp. sativa* | Landrace | NA | LC | Dioecious | [4], [15], [44] |
|  | C52 | Schurig | Germany | CAN 60 | *C. sativa ssp. sativa* | Selected line | Russian landraces | LC | Dioecious | [5], [45] |
| Hanfama GmbH | C8 | Carmagnola | Italy | NA | *C. sativa ssp. sativa var. sativa* | Landrace | NA | LC | Dioecious | [5], [29], [46] |
|  | C73 | Félina 32 | France | NA | *C. sativa ssp. sativa var. sativa* | Inbred line | (Kompolti x Fibrimon 24) x Fibrimon 24 | LC | Monoecious | [5], [47] |
|  | C72 | Férimon | France | NA | *C. sativa ssp. sativa var. sativa* | Inbred line | Fibrimon 21 | LC | Monoecious | [5], [48] |
|  | C74 | Futura 75 | France | NA | *C. sativa ssp. sativa var. sativa* | Inbred line | (Fibridia x Fibrimon 24) x Fibrimon 24 | LC | Monoecious | [5], [49] |
|  | C75 | Orion 33 | France | NA | *C. sativa ssp. sativa var. sativa* | NA | NA | LC | Monoecious | [50] |
|  | C9 | Santhica 27 | France | NA | *C. sativa ssp. sativa var. sativa* | NA | NA | LC | Monoecious | [51] |
| Hanfland GmbH | C1 | Fedora 17 | France | NA | *C. sativa ssp. sativa var. sativa* | Inbred line | (Fibrimon 21 x Jus 9) x Fibrimon 21 | LC | Monoecious | [52], [53] |
| KFU Botanical Garden | C2 | Silvana x Manitoba Poison | Romania/  South Africa | NA | *C. sativa ssp. sativa var. sativa x ssp. indica var. indica* | Segregating hybrid | Silvana x African landraces | THC-enriched | Dioecious | [54], [55], [56] |
| LVMI Silava | C70 | Purini | Latvia | LVA02775 | *C. sativa ssp. sativa* | Landrace | NA | LC | Dioecious | [57], [58], [59] |
| Sensi Seeds | C87 | Afghani #1 | Afghanistan | 1500010 | *C. sativa ssp. indica* | Landrace | NA | THC- enriched | Dioecious | [60], [61] |
|  | C86 | Hindu Kush | Afghanistan | 1500001 | *C. sativa ssp. indica* | Landrace | NA | THC- enriched | Dioecious | [61], [62] |
|  | C91 | Master Kush | Afghanistan | 1510002 | *C. sativa ssp. indica var. indica* | Selected line | Hindu Kush | THC- enriched | Dioecious | [63] |
|  | C90 | Mexican Sativa | NA | 1500038 | *C. sativa ssp. indica var. indica* | Cross hybrid | Oaxaca x Durban x Hash Pakistan | THC- enriched | Dioecious | [64] |
|  | C89 | Ruderalis Indica | NA | 1500034 | *C. sativa ssp. indica var. indica x ssp. sativa var. spontanea* | Cross hybrid | NA | CBD- enriched | Dioecious | [65] |
|  | C88 | Skunk #1 | NA | 1500024 | *C. sativa ssp. indica var. indica* | Cross hybrid | Afghani 1 x Mexican Acapulco Gold x Colombian Gold | THC- enriched | Dioecious | [15], [61], [66] |

[1] L. Grabowska, H. Burczyk, P. Baraniecki, J. Kozak, and M. Strybe, “Maintenance Breeding of Polish Hemp Cultivar Beniko,” Journal of Natural Fibers, vol. 5, no. 3, pp. 208–217, Sep. 2008, doi: 10.1080/15440470802252453.

[2] M. Szalata, M. Dreger, G. Mańkowska, M. Górska-Paukszta, M. Ożarowski, and K. Wielgus, “Determination of CBD in Ethanol Extracts Prepared from Hemp (Cannabis sativa L.) Cultivar Beniko Using Dynamic Maceration and Ultrasound Assisted Extraction,” in IECPS 2021, MDPI, Dec. 2021, p. 40. doi: 10.3390/IECPS2021-12038.

[3] Gene bank — Czechia, “Cannabis sativa L., 05X1500003,” 1995, Accessed: Jul. 10, 2023. [Online]. Available: https://grinczech.vurv.cz/gringlobal/accessiondetail.aspx?id=31364

[4] E. Small and D. Marcus, “Tetrahydrocannabinol Levels in Hemp (Cannabis sativa) Germplasm Resources,” Economic Botany, vol. 57, no. 4, pp. 545–558, 2003.

[5] E. de Meijer, “Fibre hemp cultivars: A survey of origin, ancestry, availability and brief agronomic characteristics,” vol. 2, no. 2, p. 8, 1995.

[6] G. Grassi and J. M. McPartland, “Chemical and Morphological Phenotypes in Breeding of Cannabis sativa L.,” in Cannabis sativa L. - Botany and Biotechnology, S. Chandra, H. Lata, and M. A. ElSohly, Eds., Cham: Springer International Publishing, 2017, pp. 137–160. doi: 10.1007/978-3-319-54564-6_6.

[7] J. C. Callaway, “Hempseed as a nutritional resource: An overview,” Euphytica, vol. 140, no. 1–2, pp. 65–72, Jan. 2004, doi: 10.1007/s10681-004-4811-6.

[8] Gene bank — Czechia, “Cannabis sativa L., 05X1500019,” 2009, Accessed: Jul. 10, 2023. [Online]. Available: https://grinczech.vurv.cz/gringlobal/accessiondetail.aspx?id=31380

[9] J. C. Callaway, “Hemp Seed Production in Finland,” Journal of Industrial Hemp, vol. 9, no. 1, pp. 97–103, Jun. 2004, doi: 10.1300/J237v09n01_11.

[10] “Arjan’s Haze 3 (Feminised),” Green Hourse Seeds, Netherlands. [Online]. Available: https://shop.greenhouseseeds.nl/feminised-cannabis-seeds/arjans-haze-3/

[11] “The Church CBD (Feminised),” Green Hourse Seeds, Netherlands. [Online]. Available: https://shop.greenhouseseeds.nl/feminised-cannabis-seeds/the-church-cbd/

[12] IPK Genebank, “Cannabis sativa L. subsp. sativa, CAN 62,” 2020, doi: 10.25642/IPK/GBIS/1430948.

[13] G. Bredemann, “Weitere Beobachtungen bei Züchtung des Hanfes auf Fasergehalt,” Der Züchter, vol. 22, no. 9, pp. 257–269, Sep. 1952, doi: 10.1007/BF00710361.

[14] IPK Genebank, “Cannabis sativa L. subsp. sativa, CAN 35,” 2021, doi: 10.25642/IPK/GBIS/89524.

[15] E. Schumann, A. Peil, and W. E. Weber, “Preliminary results of a German field trial with different hemp (Cannabis sativa L.) accessions,” p. 9, 1996.

[16] IPK Genebank, “Cannabis sativa L., CAN 48,” 2020, doi: 10.25642/IPK/GBIS/243619.

[17] E. P. M. de Meijer, H. J. van der Kamp, and F. A. van Eeuwijk, “Characterisation of Cannabis accessions with regard to cannabinoid content in relation to other plant characters,” Euphytica, vol. 62, no. 3, pp. 187–200, Jan. 1992, doi: 10.1007/BF00041753.

[18] IPK Genebank, “Cannabis sativa L. subsp. sativa, CAN 67,” 2021, doi: 10.25642/IPK/GBIS/2276998.

[19] F. Höppner, “Yield and quality of fibre and oil of fourteen hemp cultivars in Northern Germany at two harvest dates,” Landbauforschung Völkenrode, 2007.

[20] IPK Genebank, “Cannabis sativa L. subsp. sativa, CAN 52,” 2020, doi: 10.25642/IPK/GBIS/1430927.

[21] G. Bredemann, K. Garber, W. Huhnke, and R. von Sengbusch, “Die Züchtung von monözischen und diözischen, faserertragreichen Hanfsorten Fibrimon und Fibridia.,” Zeitschrift für Pflanzenzüchtung, no. 6, pp. 235–245, 1956.

[22] IPK Genebank, “Cannabis sativa L. subsp. sativa, CAN 44,” 2021, doi: 10.25642/IPK/GBIS/263605.

[23] IPK Genebank, “Cannabis sativa L. subsp. sativa, CAN 55,” 2020, doi: 10.25642/IPK/GBIS/1430934.

[24] IPK Genebank, “Cannabis sativa L. subsp. sativa, CAN 43,” 2021, doi: 10.25642/IPK/GBIS/263604.

[25] “The hemp seed hub: A thread for those who seek seeds and infos on hemp,” icmag. [Online]. Available: https://www.icmag.com/threads/the-hemp-seed-hub-a-thread-for-those-who-seek-seeds-and-infos-on-hemp.336827/

[26] IPK Genebank, “Cannabis sativa L. subsp. sativa, CAN 70,” 2021, doi: 10.25642/IPK/GBIS/2295802.

[27] IPK Genebank, “Cannabis sativa L. subsp. sativa, CAN 45,” 2020, doi: 10.25642/IPK/GBIS/243581.

[28] IPK Genebank, “Cannabis sativa L. subsp. sativa, CAN 66,” 2020, doi: 10.25642/IPK/GBIS/1430596.

[29] S. L. Cosentino, E. Riggi, G. Testa, D. Scordia, and V. Copani, “Evaluation of European developed fibre hemp genotypes (Cannabis sativa L.) in semi-arid Mediterranean environment,” Industrial Crops and Products, vol. 50, pp. 312–324, Oct. 2013, doi: 10.1016/j.indcrop.2013.07.059.

[30] IPK Genebank, “Cannabis sativa L. subsp. sativa, CAN 51,” 2020, doi: 10.25642/IPK/GBIS/1430925.

[31] IPK Genebank, “Cannabis sp., CAN 41,” 2020, doi: 10.25642/IPK/GBIS/262350.

[32] IPK Genebank, “Cannabis sativa L., CAN 39,” 2020, doi: 10.25642/IPK/GBIS/93421.

[33] IPK Genebank, “Cannabis sativa L., CAN 17,” 2020, doi: 10.25642/IPK/GBIS/49243.

[34] IPK Genebank, “Cannabis sativa L., CAN 40,” 2021, doi: 10.25642/IPK/GBIS/98082.

[35] IPK Genebank, “Cannabis sativa L. subsp. sativa, CAN 21,” 2021, doi: 10.25642/IPK/GBIS/49247.

[36] IPK Genebank, “Cannabis sativa L. subsp. sativa, CAN 37,” 2020, doi: 10.25642/IPK/GBIS/89596.

[37] IPK Genebank, “Cannabis sp., CAN 57,” 2020, doi: 10.25642/IPK/GBIS/1430938.

[38] IPK Genebank, “Cannabis sativa L. subsp. sativa, CAN 64,” 2021, doi: 10.25642/IPK/GBIS/224638.

[39] IPK Genebank, “Cannabis sativa L. subsp. sativa, CAN 63,” 2021, doi: 10.25642/IPK/GBIS/249874.

[40] IPK Genebank, “Cannabis sativa L. subsp. spontanea Serebr., CAN 22,” 2021, doi: 10.25642/IPK/GBIS/50060.

[41] IPK Genebank, “Cannabis sativa L., CAN 24,” 2021, doi: 10.25642/IPK/GBIS/50062.

[42] IPK Genebank, “Cannabis sativa L., CAN 19,” 2021, doi: 10.25642/IPK/GBIS/49245.

[43] IPK Genebank, “Cannabis sativa L. subsp. sativa, CAN 23,” 2021, doi: 10.25642/IPK/GBIS/82795.

[44] IPK Genebank, “Cannabis sativa L., CAN 16,” 2021, doi: 10.25642/IPK/GBIS/49242.

[45] IPK Genebank, “Cannabis sativa L. subsp. sativa, CAN 60,” 2020, doi: 10.25642/IPK/GBIS/1430944.

[46] P. Ranalli, “Current status and future scenarios of hemp breeding,” Euphytica, vol. 140, no. 1–2, pp. 121–131, Jan. 2004, doi: 10.1007/s10681-004-4760-0.

[47] “Félina 32,” Hempit, France. [Online]. Available: https://www.hemp-it.coop/en/produit/felina-32/

[48] “Férimon,” Hempit, France. [Online]. Available: https://www.hemp-it.coop/en/produit/ferimon/

[49] “Futura 75,” Hempit, France. [Online]. Available: https://www.hemp-it.coop/en/produit/futura-75/

[50] “Orion 33,” Hempit, France. [Online]. Available: https://www.hemp-it.coop/en/produit/orion-33/

[51] “Santhica 27,” Hempit, France. [Online]. Available: https://www.hemp-it.coop/en/produit/santhica-27/

[52] “Fedora 17,” Hanfland GmbH, Austria. [Online]. Available: https://www.hanfland.at/hanfland-shop/mehr-vom-hanf/hanf-gartensaatgut-500g/

[53] “Fedora 17,” HempIt. [Online]. Available: https://www.hemp-it.coop/en/produit/fedora-17/

[54] V. Tabără, C. F. Duma, and C. Moldovan, “Silvana (LV 200) - A new dioic hemp cultivar developed at the SCDA Lovrin (Timis County),” p. 6.

[55] “Manitoba Poison,” Leafly. [Online]. Available: https://www.leafly.com/strains/manitoba-poison

[56] “Manitoba Poison,” Wikileaf. [Online]. Available: https://www.wikileaf.com/strain/manitoba-poison/

[57] Latvian State Forest Research Institute “Silava” — Latvia, “Cannabis sativa L., LVA02775,” 2019, Accessed: Jul. 10, 2023. [Online]. Available: http://sesto.nordgen.org/sesto/index.php?scp=lva&thm=sesto&accnumtxt=LVA02775

[58] I. Baltiņa, Z. Zamuška, V. Stramkale, and G. Strazds, “Physical Properties of Latvian Hemp Fibres,” ETR, vol. 2, p. 237, Aug. 2015, doi: 10.17770/etr2011vol2.964.

[59] L. Poiša and L. Antipova, “The Influence of Agrotechnical Factors on Productivity Hemp (Cannabis sativa L.),” UBSRAS, vol. 26, no. 2, 2022, doi: 10.56407/2313-092X/2022-26(2)-3.

[60] “Afghani #1 Regular Seeds,” Sensi Seeds, Netherlands. [Online]. Available: https://sensiseeds.com/en/cannabis-seeds/sensi-seeds/afghani-1

[61] P. Ranalli, Ed., Advances in hemp research. New York: Food Products Press, 1999.

[62] “Hindu Kush Regular Seeds,” Sensi Seeds, Netherlands. [Online]. Available: https://sensiseeds.com/de/hanfsamen/sensi-seeds/hindu-kush

[63] “Master Kush Regular Seeds by White Label,” Sensi Seeds, Netherlands. [Online]. Available: https://sensiseeds.com/en/cannabis-seeds/whitelabel/master-kush

[64] “Mexican Sativa Regular Seeds,” Sensi Seeds, Netherlands. [Online]. Available: https://sensiseeds.com/en/cannabis-seeds/sensi-seeds/mexican-sativa

[65] “Ruderalis Indica Regular Seeds,” Sensi Seeds, Netherlands. [Online]. Available: https://sensiseeds.com/en/cannabis-seeds/sensi-seeds/ruderalis-indica

[66] “Skunk #1 Regular Seeds,” Sensi Seeds, Netherlands. [Online]. Available: https://sensiseeds.com/en/cannabis-seeds/sensi-seeds/skunk-1

**Table S2** Hyperparameters of gradient boosted-trees.

| **Number of trees** | **Minimum number of observations per node** | **Maximum tree depth** | **Learning rate** | **Minimum loss reduction to split further** | **Proportion of data used for fitting** | **Engine** |
| --- | --- | --- | --- | --- | --- | --- |
| 1000 | 8 | 8 | 0.02 | 1e-8 | 0.8 | XGBoost |

**Table S3** Statistics of the calculated alpha diversity indices described by median and interquartile range (IQR) for each *Cannabis* **(a)** genotype, **(b)** domestication grade and **(c)** chemotype in the rarefied dataset.

**a**

| **Genotype** | **Observed median** | **Observed IQR** | **Shannon median** | **Shannon IQR** |
| --- | --- | --- | --- | --- |
| C1 | 15 | 17-15 | 0,36 | 0,58-0.26 |
| C10 | 58,5 | 72,75-44 | 1,00 | 1,11-0.83 |
| C11 | 49 | 54-43 | 1,37 | 1,56-1.17 |
| C13 | 56 | 69-52 | 2,26 | 2,37-2.13 |
| C15 | 47 | 57,5-42 | 1,34 | 1,42-1.33 |
| C16 | 43,5 | 49,5-25 | 1,46 | 1,91-1.09 |
| C17 | 21 | 23-20 | 0,65 | 0,78-0.52 |
| C18 | 62 | 71-34.5 | 1,44 | 1,61-1.08 |
| C2 | 45,5 | 53,5-41 | 1,91 | 2,02-1.55 |
| C28 | 20 | 21,75-17.75 | 0,58 | 0,73-0.51 |
| C30 | 30,5 | 57-12.25 | 0,98 | 2,14-0.47 |
| C32 | 66 | 77,75-47.75 | 1,90 | 2,15-1.69 |
| C33 | 52,5 | 68-40.25 | 1,82 | 2,27-1.54 |
| C34 | 57 | 59,5-55.5 | 2,05 | 2,06-1.65 |
| C36 | 67 | 72-57 | 2,02 | 2,11-1.89 |
| C37 | 25 | 27-22 | 1,27 | 1,39-1.10 |
| C38 | 18 | 25-14 | 0,39 | 0,48-0.29 |
| C41 | 17,5 | 19,5-16.25 | 0,47 | 0,88-0.36 |
| C43 | 31 | 31-28.25 | 0,97 | 1,36-0.67 |
| C44 | 21,5 | 29,75-19 | 0,50 | 0,65-0.41 |
| C47 | 84 | 100,75-66 | 2,36 | 2,39-2.28 |
| C49 | 43 | 54-27 | 1,84 | 2,05-1.74 |
| C52 | 64 | 68,5-53.75 | 1,83 | 1,92-1.57 |
| C54 | 63 | 77-56.25 | 1,54 | 1,66-1.45 |
| C55 | 47 | 51-41 | 1,81 | 1,95-1.67 |
| C56 | 32,5 | 40,75-21 | 1,09 | 1,52-0.77 |
| C58 | 62 | 66-58 | 2,01 | 2,05-1.97 |
| C59 | 29 | 30,25-26.75 | 1,39 | 1,43-1.24 |
| C62 | 25 | 32,5-22 | 0,43 | 2,02-0.24 |
| C65 | 21 | 21-20.25 | 0,41 | 0,60-0.33 |
| C67 | 17,5 | 23,25-15.5 | 0,68 | 0,84-0.55 |
| C70 | 67 | 72,25-55.25 | 1,44 | 1,56-1.29 |
| C72 | 14 | 15,75-11.5 | 0,22 | 0,33-0.17 |
| C73 | 13,5 | 14,75-12 | 0,31 | 0,41-0.24 |
| C74 | 34 | 40-17 | 0,90 | 1,31-0.25 |
| C75 | 24,5 | 37,75-18 | 0,83 | 1,45-0.35 |
| C76 | 52,5 | 75,75-44.75 | 0,73 | 0,89-0.52 |
| C77 | 64,5 | 73,25-59.5 | 1,93 | 2,05-1.85 |
| C8 | 20,5 | 22,75-16.5 | 0,56 | 0,64-0.47 |
| C86 | 26 | 31-23.5 | 0,68 | 0,84-0.66 |
| C87 | 42 | 45-21.25 | 1,17 | 1,24-1.13 |
| C88 | 30,5 | 31-24.25 | 1,14 | 1,18-1.00 |
| C89 | 61 | 64,5-39 | 1,35 | 1,43-1.20 |
| C9 | 22 | 26,75-18 | 0,49 | 0,93-0.37 |
| C90 | 26 | 31,5-21.5 | 0,57 | 0,71-0.37 |
| C91 | 65 | 67,5-61.25 | 1,38 | 1,49-1.27 |

**b**

| **Domestication grade** | **Observed median** | **Observed IQR** | **Shannon median** | **Shannon IQR** |
| --- | --- | --- | --- | --- |
| Landraces | 38 | 58-22 | 1.13 | 1.55-0.71 |
| Selected lines | 65.5 | 74.75-60.25 | 1.83 | 2.14-1.47 |
| Cross hybrids | 27 | 38.25-19 | 1.10 | 1.46-0.5 |
| Inbred lines | 15.5 | 20.25-13 | 0.31 | 0.64-0.23 |

**c**

| **Chemotype** | **Observed median** | **Observed IQR** | **Shannon median** | **Shannon IQR** |
| --- | --- | --- | --- | --- |
| High | 45 | 62-29.5 | 1.21 | 1.55-0.83 |
| Low | 28 | 54-18 | 1.05 | 1.66-0.48 |

**Table S4** Permutational multivariate analysis (permanova, 999 permutations) with adonis2 in Vegan and Kruskal-Wallis rank sum test on *Cannabis* genotype, domestication grade and chemotype within the same provider to assess differences in beta and alpha diversity, respectively. The last row shows the differences between providers. Significant differences (α = 0.05) are marked (*).

|  | | **permanova** | | | | | **Kruskal-Wallis rank sum test** | | | | | |
| --- | --- | --- | --- | --- | --- | --- | --- | --- | --- | --- | --- | --- |
|  |  |  |  |  |  |  | **Observed Richness** | | | **Shannon *H’* Index** | | |
| **Provider** | **Feature** | **df** | **SumOfSqs** | **R^2^** | **F** | **Pr(>F)** | **chi-squared** | **df** | **p-value** | **chi-squared** | **df** | **p-value** |
| **IPK** | Genotype | 26 | 26.148 | 0.48451 | 8.5676 | ≤ 0.001***** | 130.91 | 26 | ≤ 0.001***** | 127.92 | 26 | ≤ 0.001***** |
|  | Domestication | 2 | 3.645 | 0.09535 | 9.7493 | ≤ 0.001***** | 33.732 | 2 | ≤ 0.001***** | 24.326 | 2 | ≤ 0.001***** |
| **CRI** | Genotype | 1 | 0.2632 | 0.12778 | 2.4904 | 0.003***** | 0.87048 | 1 | 0.3508 | 2.6471 | 1 | 0.1037 |
| **Hanfama GmbH** | Genotype | 5 | 2.5668 | 0.31932 | 5.0664 | ≤ 0.001***** | 23.52 | 5 | ≤ 0.001***** | 12.575 | 5 | 0.02771***** |
| **Green House Seeds Co.** | Genotype | 1 | 0.7065 | 0.20108 | 4.5305 | ≤ 0.001***** | 1.2023 | 1 | 0.2729 | 13.72 | 1 | ≤ 0.001***** |
| **Sensi Seeds** | Genotype | 5 | 2.6893 | 0.29494 | 4.3505 | ≤ 0.001***** | 29.497 | 5 | ≤ 0.001***** | 29.63 | 5 | ≤ 0.001***** |
|  | Domestication | 2 | 1.2366 | 0.13562 | 4.3145 | ≤ 0.001***** | 17.383 | 2 | ≤ 0.001***** | 12.258 | 2 | ≤ 0.001***** |
| **Total** | | 7 | 15.548 | 0.17053 | 13.011 | ≤ 0.001***** | 114.36 | 7 | ≤ 0.001***** | 98.754 | 7 | ≤ 0.001***** |

**Table S5** Identity and alignment report between identified biomarker ASVs and isolated bacteria from *Cannabis* seed endosphere.

| **Illumina** | | | **Sanger** | | | | **NCBI BLAST+ blastn tool** | | |
| --- | --- | --- | --- | --- | --- | --- | --- | --- | --- |
| **ASV** | **Seq Length** | **Silva Taxonomy** | **Isolate** | **Seq Length** | **NCBI Taxonomy** | **Identity (%)** | **Match (%)** | **Coverage (%)** | **Bit score** |
| 3151 | 234 | *Pseudomonas putida* | C2813 | 1140 | *Pseudomonas punonensis* | 99.64% | 100% | 100% | 433 |
| 3140 | 234 | *Pseudomonas sp.* | C6532 | 1061 | *Pseudomonas congelans* | 100% | 100% | 100% | 433 |
| 3007 | 234 | *Bacillus sp.* | C17A71 | 1083 | *Niallia circulans* | 99.72% | 100% | 100% | 433 |
| 4090 | 234 | *Rathayibacter sp.* | C37B11 | 1048 | *Rathayibacter festucae* | 100% | 100% | 100% | 433 |
| 3055 | 234 | *Bacillus sp.* | C1141 | 1453 | *Peribacillus frigotolerans* | 99.72% | 100% | 100% | 450 |

**Table S6** Top 25 gene features found in *Bacillus frigotolerans* C1141 genome, regarding the number of associated genes.

| **Gene feature** | **PGPT Function** | **Genes and gene clusters** | **Total** |
| --- | --- | --- | --- |
| 1. Purine metabolism | Substract usage | ade, adk\|AK, aIIC-D, apt, cusC, deoB, dgk, gmk, guaA-C, hit, hprT\|hpt, iunH, nudFm pgm, prsA, pucG, purA-F, purH, purK-N, purQ, pur S-T, pyk, sat\|met3, spot, tilS_hprT, urea-C, xpt, yjjX, yIbA, ywaC\|yjbM | 44 |
| 1. Pyrimidine metabolism | Substract usage | carA-B, cdd, cmk, comEB\|tadA, dck, deoD, ndk, nrdA\|nrdE, nrdB\|nrdF nucA\|ushA\|yhcR\|yfkN, pdp\|deoA, punA, pyrB-C, pyrDI, pyrDII, pyrE-H, tdk, thyA, tmk, udk, upp, ydfG, yfkN, ymdB | 29 |
| 1. Halotolerance-related enzymes | Abiotic stress neutralization | abIA, acrR1, arsC, arsC1, atpA-H, dps\|dpsA, Ion, mtnN\|pfs\|yadA, nadA, prkA\|yeaG, rpmEB, rsbV, rseP, rspB, smpB, spot, ycaD, yciG\|ymdF\|gsiB, yjbC, ykgA, ytaB | 28 |
| 1. Vitamin B9 or foliate metabolism | Abiotic stress neutralization | fmt, foIA-E, foIE2, foIK, foIP, gcvT, glyA, metH, pabBC, phoA, phoD, purH, purN, purT-U, ribA, ribBA, thyA, ygfA\|fthC\|yqgN\|folN, yitJ | 24 |
|  | Germination stimulation |  |  |
|  | Plant vitamin production |  |  |
| 1. Tricarboxylic acid cycle | Substract usage | aarC\|cat1, aceF\|pdhC, acnA, CSgltA, frdA, fumA\|fumB, fumC, icd, korA\|oorA\|oforA, korB\|oorB\|oforB, lpd\|pdhD, mdh, mqo, pckA, pdhA-B, pyc, sdhA\|fdrA, sdhB\|fdrB, sdhC\|fdrC, sucA-D | 24 |
| 1. Stress response proteins | Stress signalling proteins | aldH\|dhaS, csbA, csbB\|gtrB\|yfdH, csbC, dps\|dpsA, gabD, ispD, ispF, nhaX, prkA\|yeaG, pyrF, sigM\|rpoE, ydaG, ydaM, ydhK, yfkH, yfkM\|pfpI\|yraA, yjgD, ykgB\|pgl, yqhQ, ytxH, ytxJ, yugU, yvgN | 24 |
| 1. Pyruvic acid biosynthesis | Phosphate solubilization | cuyA, dapA\|mosA, dat, dgaF, dld, dsdA, eda, hpaF, ilvA\|tdcB, ligK\|galC, maeA\|sfcA\|ywkA, mccB, metC, mhpE, nagK, patB\|maIY, ptsI, pyk, sdaA\|sdaB\|tdcG, sseA, tpav\|bioA\|yhxA, ttuC\|dmlA, yjhH\|yagE | 23 |
|  | Potassium solubilization |  |  |
| 1. Peptide metabolism | Substract usage | acdP, ddpA\|ABC_PE_S, ddpB\|appB, ddpC\|appC, ddpD, ddpF, dppC1, map, nprM, opaA\|pepQ, oppA\|mppA, oppB, oppD, oppF, pepD, pepF\|pepB, pepP, pepS\|amp\|ampT, pepT, TC_POT, vpr, ykfC, ywaD | 23 |
| 1. Vitamin B12 or cobalamin biosynthesis | Plant vitamin production | cbiA, cbiB\|cobD, cbiC-D, cbiF-G, cbiH60, cbiJ, cbiL, cbiXm cobA\|btuR, cobC\|phpB, cobD, cobL, cobP\|cobU, cobQ\|cbiP, cobS\|cobVm cobU\|cobT, rhnA_cobC, sirA\|ylnD\|cysG\|cobA | 20 |
| 1. Vitamin B5 or pantothenic acid co-factor biosynthesis | Plant vitamin production | LYS5\|acpT, UPB1_like\|pydC, acpS, budB\|ilvK\|alsS\|ilvB\|ilvG\|ilvI, coaA, coaBC\|dfp, coaD\|kdtB, coaE, dht\|hydA, ilvC-E, ilvH\|ilvN, panB-D, panE\|apbA, preA, preT | 19 |
| 1. Multidrug efflux genes | Bacterial fitness | bcr\|tcaB, vlt, ebrA-B, efrA-B, emrB, emrR\|mprA, lmrB, mdeA, mdlA\|smdA, mdlB\|smdB, mdtG, mta\|ywnD, TC_MATE\|norM\|mdtK\|dinF, TC_SMR3, ydhP, ykkC, ymfE | 19 |
| 1. Spore germination protein | Spore production | gerAB, gerAC, gerBA, gerD, gerKA-C, gerPB-C, gerPE-F, gerQ, yaaH, yfkQ-R, yndD-F, ypeB | 19 |
| 1. Biofilm regulators | Quorum sensing or biofilm formation | abrB, epsE, flgM, fliA\|sigD\|whiG, hsbR, icaR, luxS, lytR\|ypdB\|yehT, lytS\|ypdA\|yehU, resE, rpoE\|sigW, sigH\|sigG\|sigF\|sigE, sinR, spo0A, spot, syIA, ycbA\|glnK, yddV\|ydeH\|yeaP | 18 |
| 1. Vitamin B1 or thiamin biosynthesis | Plant vitamin production | adk\|AK, dxs, nifS\|iscS, phoA, rsgA\|engC, tenA, tenI, thiC-G, thiI-J, thiM, thiN\|TPK1\|THI80, thiO | 17 |
|  | ISR |  |  |
| 1. HEME or SIRO biosynthesis | Plant vitamin production | ahbD, cobA_hemD, ctaA, ctaB\|cyoE, gltX, hemA-E, hemH\|ywfl, hemL, hemN\|hemZ, hemQ, hemY, sirA\|ylnD\|cysG\|cobA, sirB | 17 |
| 1. Volatile-related fatty acid metabolism | Biotic stress neutralization | accA, accB\|bccP, accC-D, acd, desA, fabD\|bmyD, fabF, fabH-I, fabL, fabZ, fadB, fadD, fadN, ymfI\|fabG\|efpI | 16 |
|  | Volatile metabolism |  |  |
| 1. Oxidative stress signalling | Quorum sensing or biofilm formation | ABC_SP_A\|ydcT, ABC_SP_P1\|ydcU, ABC_SP_P\|ydcV, ABC_SP_S\|ydcS, oxyR, paiA, patA1, potA-D, pup, speB, speE\|SRM\|SPEC_3\|SPSD, speG, spuC | 16 |
| 1. Fatty acid degradation | Substract usage | acd, adhP, aldH\|dhaS, atoA, atoC, atoD, bcd, cpxB, ECHS1, fadB, fadD, fadN, frmA\|ADH5\|adhC, HADH_like, paaF\|echA, yiaY | 16 |
| 1. Glycoside glycosidases or glycosylhydrolases | Substract usage | acm, amyA\|malS, bcsZ\|wssD\|yhjM, beta_amylase, bgIA, cd\|ma\|npIT, celF\|licH\|chbF, EC_3_2_1_205, fruA, lytD\|lytB, mail, nagZ, pulA, sac\|levB, treC, treZ\|glgZ | 16 |
| 1. Paraquat stress reduction | Abiotic stress neutralization | aldH\|dhaS, clsA_B\|ybhO\|ywiE, clsC\|ymdC, csbA, csbC, ydaG, ydhK, yerD, yfkH, yflA\|TC_AGCS, yitT, yjbC, yjgD, ylxP, yqhQ, yugU | 16 |
| 1. Sodium transport | Abiotic stress neutralization | gltS, mnhA-G, ndaC, nhaK\|TC_CPA1, panS\|yocS\|ybaS, putP\|ycgO, TC_DAACS, TC_NSS, TC_SSS\|yerK\|opuE, yjbB | 16 |
| 1. Spermidine or putrescine metabolism | Abiotic stress neutralization | ABC_SP_A\|ydcT, ABC_SP_P\|ydcV, ABC_SP_P1\|ydcU, ABC_SP_S\|ydcS, paiA, patA1, potA-D, pup, speB, speE\|SRM\|SPEC_3\|SPSD, speG, spuC | 15 |
|  | Biotic stress neutralization |  |  |
| 1. Vitamin B3 or niacin biosynthesis | Abiotic stress neutralization | deoD, iunH, nadA-E, nadE2, nadX, nucA\|ushA\|yhcR\|yfkN, pncA-C, punA, yfkN | 15 |
|  | Root colonization |  |  |
| 1. Proline metabolism | Abiotic stress neutralization | argB-C, argD\|pqqI, argE, argHA, argJ, gbuA\|proV, gbuC\|proX, ocd, proA-C putP\|ycgO, TC_SSS\|yerK\|opuE, yobR | 15 |
| 1. IAA related tryptophan metabolism/pathway | Abscisic acid degradation | lysN, patA, patA1, soIA, tpav\|bioA\|yhxA, trpA-D, trpE\|phnA, trpF, trpG\|phnB, trpS, yodT, yugH | 15 |
|  | Branching stimulation |  |  |
